# Elucidation of the biosynthesis of carnosic acid and its reconstitution in yeast

**DOI:** 10.1038/ncomms12942

**Published:** 2016-10-05

**Authors:** Ulschan Scheler, Wolfgang Brandt, Andrea Porzel, Kathleen Rothe, David Manzano, Dragana Božić, Dimitra Papaefthimiou, Gerd Ulrich Balcke, Anja Henning, Swanhild Lohse, Sylvestre Marillonnet, Angelos K. Kanellis, Albert Ferrer, Alain Tissier

**Affiliations:** 1 Department of Cell and Metabolic Biology, Leibniz Institute of Plant Biochemistry, Weinberg 3, Halle 06120, Germany; 2 Department of Bioorganic Chemistry, Leibniz Institute of Plant Biochemistry, Weinberg 3, Halle 06120, Germany; 3 Program of Plant Metabolism and Metabolic Engineering, Centre for Research in Agricultural Genomics, Campus UAB, 08193 Bellaterra, Spain; 4 Faculty of Pharmacy, Department of Biochemistry and Molecular Biology, University of Barcelona, 08028 Barcelona, Spain; 5 Group of Biotechnology of Pharmaceutical Plants, Laboratory of Pharmacognosy, Department of Pharmaceutical Sciences, Aristotle University of Thessaloniki, 54124 Thessaloniki, Greece

## Abstract

Rosemary extracts containing the phenolic diterpenes carnosic acid and its derivative carnosol are approved food additives used in an increasingly wide range of products to enhance shelf-life, thanks to their high anti-oxidant activity. We describe here the elucidation of the complete biosynthetic pathway of carnosic acid and its reconstitution in yeast cells. Cytochrome P450 oxygenases (CYP76AH22-24) from *Rosmarinus officinalis* and *Salvia fruticosa* already characterized as ferruginol synthases are also able to produce 11-hydroxyferruginol. Modelling-based mutagenesis of three amino acids in the related ferruginol synthase (CYP76AH1) from *S. miltiorrhiza* is sufficient to convert it to a 11-hydroxyferruginol synthase (HFS). The three sequential C20 oxidations for the conversion of 11-hydroxyferruginol to carnosic acid are catalysed by the related CYP76AK6-8. The availability of the genes for the biosynthesis of carnosic acid opens opportunities for the metabolic engineering of phenolic diterpenes, a class of compounds with potent anti-oxidant, anti-inflammatory and anti-tumour activities.

## Abstract (web summary)

Diterpenes are plant products with high antioxidant properties and potential application as food additives and therapeutics. Here, the authors describe the complete biosynthetic pathway of carnosic acid and reconstruct it in yeast, opening the way to metabolic engineering of phenolic diterpenes.

## Graphical abstract

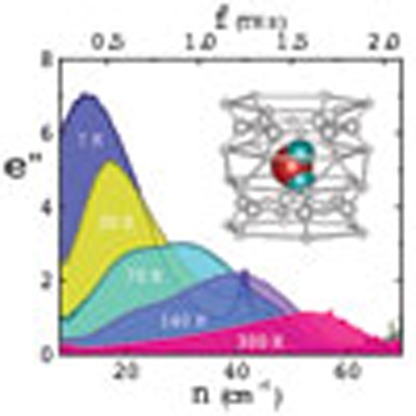

Terpenoid-based drugs such as taxol and artemisinin have become indispensable in the treatment of cancer and infectious diseases^1^,^2^. In addition, belonging to the large family of terpenoids are the phenolic labdane-type diterpenes carnosic acid (CA), carnosol (CO) and pisiferic acid (PA; Fig. 1) from rosemary (*Rosmarinus officinalis*) and various sage species (*Salvia sp*.). These compounds show diverse biological and chemical activities, including anti-oxidative, anti-cancer, anti-inflammatory, as well as anti-microbial properties, and have been proposed as preventive agents and treatments for neurodegenerative disorders^3^,^4^,^5^,^6^,^7^,^8^,^9^. However, the major use of CA and CO is based on their strong antioxidative properties, which make them highly relevant and approved food additives in Europe, China and Japan^10^. Furthermore, CA has been proposed as the precursor of a whole range of diterpenoids including the tanshinones from *Salvia miltiorrhiza* that are being actively investigated for their anti-cancer activities^11^. The first committed steps in the biosynthesis of this group of diterpenes are catalysed by the terpene synthases copalyl diphosphate synthase (CPS) and a kaurene synthase-like enzyme called miltiradiene synthase (MiS). CPS and MiS cyclize geranylgeranyl diphosphate (GGPP) to copalyl diphosphate and copalyl diphosphate to miltiradiene, respectively^12^ (Fig. 1). The oxidation of miltiradiene to abietatriene occurs spontaneously, but can be accelerated by exposure to ultraviolet irradiation^13^,^14^. One key intermediate in the downstream pathway is ferruginol. Several cytochrome P450 monooxygenases (CYP) of the CYP76 clan from *S. miltiorrhiza*, *S. fruticosa* and *R. officinalis*, which are able to convert abietatriene to ferruginol, have been characterized^14^,^15^,^16^. These enzymes are collectively called ferruginol synthases (FSs). *In vitro* characterization of one of these enzymes (CYP76AH4 from *R. officinalis*) indicated that abietatriene rather than miltiradiene is the substrate^14^. We show here that the previously identified FS from *S. fruticosa* and *R. officinalis* carry out not a single but two successive oxidations leading to the next intermediate 11-hydroxyferruginol and were renamed 11-hydroxyferruginol synthases (HFSs). Taking advantage of the similarity between these HFSs and the FS, we show that the exchange of three amino acid residues in the FS from *S. miltiorrhiza* by the corresponding residues in HFS is sufficient to convert the FS to a HFS. We also identify a group of related CYPs from *R. officinalis* and *S. fruticosa* with C_20_-oxidase (C_20_Ox) activity whose co-expression in yeast with the diterpene synthases and HFS leads to the production of CA, itself spontaneously oxidizing to CO.

## Results

### Identification of HFSs

**Figure 2:**
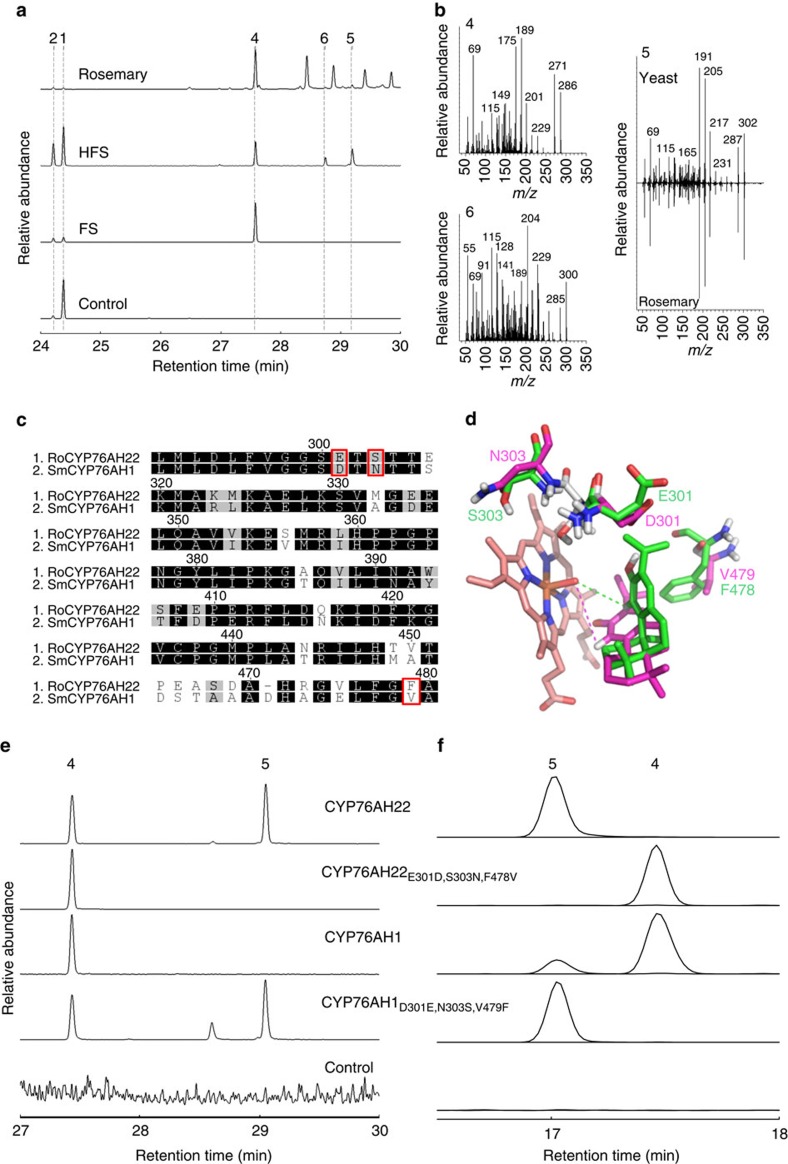
Homology modelling-based mutagenesis of FS and HFS. (**a**) Part of the GC–MS profile of hexane extracts from rosemary leaf surfaces and from yeast strains co-expressing GGPPS, CPS, MS, ATR1 and indicated CYPs (selected *m/z* signals: 270, 272, 286, 300 and 302). Miltiradiene (1), abietatriene (2), ferruginol (4), 11-hydroxyferruginol (5) and hydroxyferruginol quinone (6). (**b**) Electron impact mass spectra of ferruginol (4), 11-hydroxyferruginol (5), which was extracted from rosemary or yeast cultures, and of hydroxyferruginol quinone (6). (**c**) Excerpts of the aligned amino acid sequences of CYP76AH1 and CYP76AH22 with the residues that were mutagenized indicated by red rectangles. (**d**) Models of the active site of CYP76AH22 (green) and CYP76AH1 (magenta) with bound ferruginol and haem (orange carbon atoms). The models were generated using 2HI4 (structure of human CYP1A2) as a template. Only in the case of CYP76AH22 can the hydrogen atom at C11 be abstracted by the reactive oxygen atom, thanks to the short distance of 3.1 Å (green dashed line). In contrast, the corresponding distance in CYP76AH1 is 4.6 Å (magenta dashed line), which is too large to support oxidation at this position. (**e**) GC–MS profile (selected *m/z* signals: 286, 300 and 302) and (**f**) LC–MS profile (selected *m/z* signals: 285.221 and 301.217) of yeast strains expressing GGPPS, CPS, MS, ATR1 and indicated CYP enzymes (wild type or mutagenized). The region of the chromatograms with signals for ferruginol (4) and 11-hydroxyferruginol (5) is shown.

Using a Golden Gate modular cloning vector system developed for yeast (see Methods), we first reconstituted ferruginol biosynthesis in yeast using CYPs previously characterized as FSs, namely CYP76AH1, CYP76AH4, CYP76AH22, CYP76AH23 or CYP76AH24 (Supplementary Fig. 1 and Supplementary Table 1)^14^,^15^,^16^. Each of these P450s was coupled with the upstream biosynthetic enzymes (CPS and MiS), the CYP reductase ATR1 (ref. 17) and a GGPP synthase (GGPPS), the latter to allow for sufficient supply of GGPP. We call this group of enzymes the core module (CM) for the production of the diterpene precursors. To ensure efficient co-expression of all genes, we developed a library of synthetic galactose inducible promoters, which are not repressed by glucose and whose members conferring the strongest expression were selected (see Methods and Supplementary Table 2). As expected, extracts of all obtained yeast strains (Supplementary Table 3) revealed the presence of miltiradiene, abietatriene and ferruginol in gas chromatography–mass spectrometry (GC–MS) measurements (Fig. 2a and Supplementary Figs 3 and 4). However, expression of either one of CYP76AH4, CYP76AH22, CYP76AH23 or CYP76AH24 resulted in the production of a new compound, which could only be found in traces when expressing CYP76AH1 (Supplementary Figs 3–5). This product was also present in small quantities in leaf surface extracts of *R. officinalis* and *S. fruticosa*. The product was purified from yeast after shake-flask expression of CYP76AH22 with the CM (Table 1) and nuclear magnetic resonance (NMR) analysis established its identity as 11-hydroxyferruginol (Supplementary Table 4)^18^. Thus, CYP76AH4, CYP76AH22, CYP76AH23 and CYP76AH24, which have been reported to be FSs, are in fact able to carry out a second hydroxylation and are therefore HFSs. The activity of FS and HFSs was confirmed by *in vitro* assays with microsomal fractions from yeast strains expressing CYP76AH1 and CYP76AH22, respectively (Supplementary Figs 6 and 7).

During NMR analysis, we noticed increasing signals that did not belong to 11-hydroxyferruginol. To check whether these signals are due to instability of 11-hydroxyferruginol, the sample was stored at −20 °C for 24 h and analysed again. Based on these specific signals, this additional compound was identified as hydroxyferruginol quinone (Supplementary Table 4 and Supplementary Fig. 8). Consequently, 11-hydroxyferruginol spontaneously auto-oxidized to the corresponding quinone^18^. This was further supported by the simultaneous detection of 11-hydroxyferruginol and hydroxyferruginol quinone in all yeast strains expressing the various HFSs (Supplementary Fig. 3). Such redox conversions of the catechol group to a quinone have been shown for CA and its derivatives and are thought to be the main reason for their high antioxidant activity^4^,^19^,^20^,^21^,^22^,^23^.

### Triple-mutants of FS recapitulate HFS activity

To analyse the functional differences between FS and HFS, we applied homology modelling to CYP76AH1, CYP76AH4, CYP76AH22, CYP76AH23 and CYP76AH24. The high degree of sequence identity between these proteins (on average 80%) resulted in predicted tertiary structures that were almost identical (see Supplementary Fig. 9 as an example). These models allowed us to identify the amino acid residues that are likely to be involved in the binding of the substrates abietatriene or ferruginol. In all enzymes the docking arrangement of abietatriene supports the oxidation reaction leading to ferruginol due to the short distance between the reactive oxygen atom bound to the haem and the proton at C12 required for activation (Supplementary Fig. 10). Comparative analysis of the three-dimensional models led to the identification of three amino acids in the active site of HFS (E301, S303 and F478), which are different in FS (D301, N303 and V479), and might be associated with the oxidation of ferruginol (Fig. 2c,d and Supplementary Fig. 10). In addition, two amino acids of FS (A117 and S118) are located in a α-helix, whereas in HFS the corresponding Gly–Gly sequence adopts a coil conformation. Although both residues are rather far away from the active site, the small conformational changes may also potentially influence the activity of the enzyme (Supplementary Figs 11 and 12). All reciprocal single mutations as well as several double, triple and quadruple mutant combinations were tested in yeast *in vivo* co-expression assays with the CM (Supplementary Figs 13 and 14). Mutations at position 117 and 118 in HFS prevented the oxidation of ferruginol, but the reciprocal mutations in FS did not support the oxidation of ferruginol, indicating that these residues are not directly required for the oxidation of ferruginol. In contrast, the double mutant FS_D301E,N303S_ could produce hydroxyferruginol, but in smaller proportions compared with HFS. Furthermore, a triple mutant FS_D301E,N303S,V479F_ had a profile indistinguishable from HFS (Fig. 2e,f), which was also confirmed by *in vitro* enzyme assays (Supplementary Figs 6 and 7). The relevance of these three amino acids in the oxidation of ferruginol is confirmed by the analysis of three-dimensional models with ferruginol docked into the active site (Fig. 2d and see Supplementary Fig. 10 for details). In the HFSs (CYP76AH4, CYP76AH22, CYP76AH23 and CYP76AH24), F478 interacts with ferruginol, allowing its correct positioning for further oxidation. In contrast, in CYP76AH1, the hydrophobic interaction between the substrate and the smaller side chain of V479 at the equivalent position combined with the repulsive interaction of the carboxylic acid moiety of D301 with the isopropyl group of ferruginol, causes the adoption of a slightly different docking arrangement. In this configuration, the hydrogen atom at C11 of ferruginol is located 4.6 Å from the reactive oxygen, a distance too large to support oxidation (Supplementary Fig. 10c). Strikingly, the carboxylic moiety of E301 in HFS is involved in a salt bridge with R481 and is thus preventing the repulsive interaction with the isopropyl group of ferruginol. On the contrary, this positioning of E301 favours hydrophobic interactions via the ethylene moiety of its side chain with the isopropyl group of ferruginol. Both exchanges combined, V479/F478 and D301E, cause a slightly different docking orientation of ferruginol in HFS with a short distance (3.1 Å) of the substrate C11 to the reactive oxygen atom bound to the heme, thereby allowing the catalytic oxidation. These two exchanges (D301E and V479/F478) are the most important ones, whereas from the model only very minor structural alterations of the N303S substitution to the docking poses and therefore to the related catalysis can be observed.

Altogether, our results point to the essential contribution of amino acid residues at positions 301, 303 and 478 for the 11-hydroxylation of ferruginol in HFS and, remarkably, the reciprocal exchange of the corresponding residues in FS recapitulated the HFS activity. Interestingly, CYP76AH3, an enzyme from *S. miltiorrhiza* that was recently shown to hydroxylate ferruginol at C11, has the same residues at these three positions as the HFS identified here^24^.

### C_20_ oxidation by CYP76AK6-8 leads to CA

**Figure 3:**
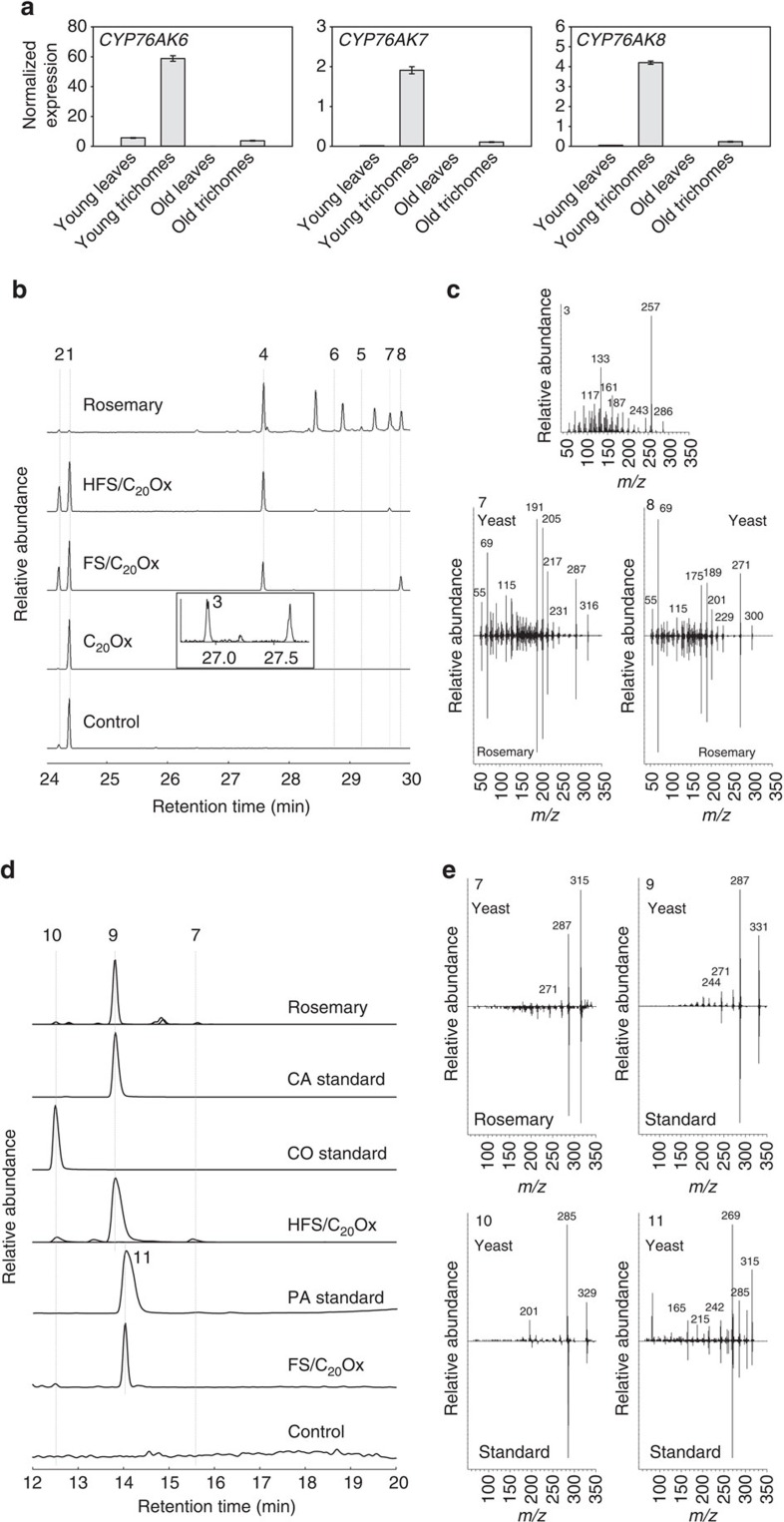
Functional analysis of C_20_Ox. (**a**) Expression profile of *CYP76AK6-8* in young and old trichomes and leaves without trichomes. *CYP76AK6* is from *S. fruticosa*, *CYP76AK7-8* are from *R. officinalis*. Quantitative reverse transcriptase–PCR data were obtained from three technical replicates and normalized to the eukaryotic elongation factor 4a. (**b**) GC–MS analysis (selected *m/z* signals: 270, 272, 286, 300, 302 and 316) of extracts from rosemary leaf surfaces or from yeast strains co-expressing GGPPS, CPS, MS, ATR1, CYP76AH22 (HFS) and CYP76AK6 (C_20_Ox). The labelled peaks correspond to miltiradiene (1), abietatriene (2), ferruginol (4), 11-hydroxyferruginol (5), hydroxyferruginol quinone (6), carnosaldehyde (7) and pisiferal (8). The framed chromatogram is part of the GC–MS analysis (selected *m/z* signal: 286) of yeast expressing GGPPS, CPS, MS, ATR1 and C_20_Ox. The peak labelled (3) corresponds to miltiradien-20-al. (**c**) Electron impact mass spectra of PDs from yeast or rosemary. The spectra were isolated from yeast strains expressing GGPPS, CPS, MS, ATR1 and CYP76AK6 for miltiradien-20-al (3); GGPPS, CPS, MS, ATR1, CYP76AH22 and CYP76AK6 for carnosaldehyde (7); and GGPPS; CPS, MS, ATR1, CYP76AH1 and CYP76AK6 for pisiferal (8). (**d**) LC–MS analysis (selected *m/z* signals: 301.217, 315.196, 331.191 and 329.175) of rosemary extract, authentic standards and yeast extracts from strains expressing GGPPS, CPS, MS, ATR1 CYP76AH22 (HFS) and CYP76AK6 (C_20_Ox). Carnosaldehyde (7), CA (9), CO (10) and PA (11). (**e**) ESI mass spectra of PDs from yeast (expressing GGPPS, CPS, MS, ATR1, CYP76AH22 and CYP76AK6 for carnosaldehyde (7); GGPPS, CPS, MS, ATR1, CYP76AH22 and CYP76AK6 for CA (9) and CO (10); and GGPPS, CPS, MS, ATR1, CYP76AH1 and CYP76AK6 for PA (11) or rosemary and authentic standards.

To identify the missing steps in the pathway to CA, we searched for genes with a similar expression pattern to those of the upstream steps, namely *CPS* and *MiS*, which are preferentially expressed in trichomes on young leaves^12^,^16^. We focused on CYP-encoding genes, as those are frequently involved in the oxidation of terpenes^25^. Real-time PCR analysis of candidate genes identified from RNA-sequencing data of *R. officinalis* and *S. fruticosa* was performed with RNA including isolated trichomes and leaves free from trichomes, as well as young and old leaves from *R. officinalis* and *S. fruticosa*. *CYP* genes from *R. officinalis* (*RoCYP76AK7* and *RoCYP76AK8*) and *S. fruticosa* (*SfCYP76AK6*) encoding highly similar proteins (Supplementary Fig. 2) showed increased transcript levels in young leaves compared with old leaves and were expressed specifically in trichomes (Fig. 3a). Interestingly, those CYPs showed high sequence similarity to the FS and HFSs.

**Figure 4:**
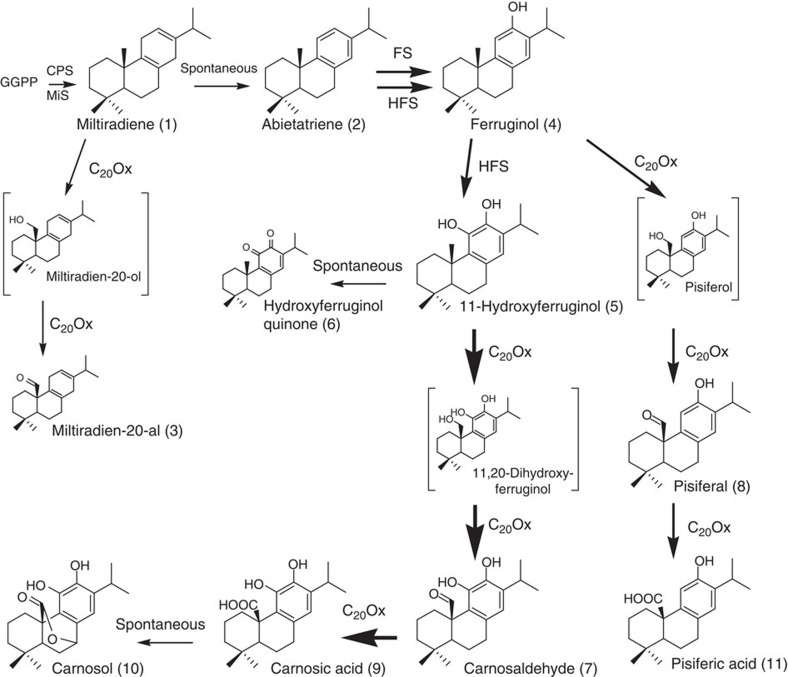
Overview of the engineered CA pathway. FS (CYP76AH1) oxidizes abietatriene to ferruginol, whereas HFS (CYP76AH22-24) are able to further oxidize ferruginol to 11-hydroxyferruginol. 11-hydroxyferruginol spontaneously oxidizes to hydroxyferruginol quinone. The C_20_Ox (CYP76AK6-8) ensure conversion of 11-hydroxyferruginol to CA, which itself spontaneously oxidizes to carnosol. C_20_Ox can also oxidize miltiradiene and ferruginol, the latter leading to PA, albeit with much lower efficiency, indicating 11-hydroxyferruginol is their preferred substrate. The size of the arrows illustrates non-quantitatively the efficiency of the corresponding reactions.

As the precise order of the oxidations in the CA pathway was not known, we first tested these CYPs by transient co-expression with CPS and MiS in *Nicotiana benthamiana*. Surface extracts of leaves expressing CPS, MiS, ATR1 and CYP76AK7 analysed by GC–MS contained a new product with a molecular mass of 286 and a base peak of 257, suggesting the presence of a diterpene with an aldehyde group (Fig. 3b,c and Supplementary Fig. 15a). Co-expression of GGPPS, CPS, MiS, ATR1 and CYP76AK6, CYP76AK7 or CYP76AK8 in yeast led to the formation of the same compound (Supplementary Fig. 15b). After purification and NMR analysis, this product was identified as miltiradien-20-al (Supplementary Table 5), thus providing first evidence that CYP76AK6, CYP76AK7 and CYP76AK8 can oxidize miltiradiene at the C20 position and are thus named C_20_Ox (Fig. 4). Although miltiradien-20-ol is the expected intermediate, it could not be detected, indicating its complete conversion to miltiradien-20-al.

The relatively low conversion of miltiradiene to miltiradien-20-al by the C_20_Ox enzymes suggested that ferruginol or 11-hydroxyferruginol rather than miltiradiene are the preferred substrates. We therefore co-expressed C_20_Ox together with HFS and the CM in yeast. GC–MS analysis of hexane extracts revealed a novel compound, which was also detected in leaf surface extracts of *R. officinalis* and *S. fruticosa* (Fig. 3b,c and Supplementary Fig. 16). This compound was purified from rosemary plants, analysed by NMR and identified as carnosaldehyde (Supplementary Table 6). As CYP enzymes are known to carry out multiple oxidations on terpenes, for example, in the biosynthesis of abietic acid (CYP720B1) or artemisinic acid (CYP71AV1)^26^,^27^, the possibility that the C_20_Ox also convert this aldehyde to a carboxylic acid was considered. However, carboxylic acids, such as CA are not detectable by GC–MS. Liquid chromatography–MS (LC–MS) analysis demonstrated that CA was indeed formed in our strains (Fig. 3d,e and Supplementary Figs 4 and 17) and was even converted to CO, which is in agreement with the literature^19^,^21^,^23^,^28^. Although CA has been reported to be spontaneously degraded to CA quinone or to epirosmanol, rosmanol and 7-methyl-epirosmanol upon stress conditions^29^,^30^, none of these compounds were detected in our experiments. In coupled *in vitro* assays with microsome fractions from strains expressing CYP76AH22 and CYP76AK8, complete conversion of 11-hydroxyferruginol to CA could be observed (Supplementary Figs 6 and 7). In addition, as in yeast engineering experiments, CO could also be detected, but not the other oxidation products normally associated with non-enzymatic oxidation of CA. Control incubations of CA with single CYP or with an empty vector control did not result in increased formation of CO, further supporting that this conversion is non-enzymatic (Supplementary Fig. 7).

To investigate whether C_20_Ox can, in addition to 11-hydroxyferruginol and miltiradiene, also oxidize ferruginol, the newly identified enzymes were co-expressed with CPS, MS and FS (CYP76AH1). GC–MS analysis and comparative analysis with published data^31^ revealed the presence of pisiferal in low amounts, indicating that indeed ferruginol is also a substrate of the C_20_Ox (Fig. 3b,c and Supplementary Figs 4 and 18). As for CA, PA could be found by LC–MS measurements (Fig. 3d,e). As CYP76AH1 is able to produce traces of 11-hydroxyferruginol, CA also co-accumulated with PA, albeit at much lower levels than with HFS (Supplementary Fig. 19), whereas CO and carnosaldehyde were not detected. Interestingly, co-expression of HFS and C_20_Ox did not result in the formation of pisiferal and PA, although ferruginol and 11-hydroxyferruginol are still detectable. This indicates that C_20_Ox preferentially uses 11-hydroxyferruginol as substrate.

### *In vivo* quantification of pathway products by NMR spectroscopy

To evaluate the respective activities of the enzyme *in vivo*, we performed absolute quantification by NMR of the intermediates and products of the pathway from yeast strains expressing different enzyme combinations (Table 1 and Supplementary Figs 20 and 21). NMR was chosen as a quantification method because of the lack of appropriate standards for 11-hydroxyferruginol, the different responses of GC–MS and LC–MS to ferruginol and 11-hydroxyferruginol, and the possibility to quantify compounds even without a standard (Methods). A strain expressing only the diterpene synthases produces miltiradiene at 5.86±1.52 μmol l^−1^. When the CYP enzymes are co-expressed with the diterpene synthases, comparable or even slightly larger amounts of the end products are produced (6.37±0.55 μmol l^−1^ of ferruginol for FS, 4.18±0.48 μmol l^−1^ of 11-hydroxyferruginol for HFS and 8.26±1.64 μmol l^−1^ of CA for HFS+C_20_Ox). Whatever the CYP enzymes that are co-expressed, miltiradiene can still be detected, albeit in significantly smaller amounts compared with the CPS/MS strain. The residual presence of miltiradiene could be explained by the extreme hydrophobicity of this compound, suggesting it could be sequestered in the membrane fraction and thereby preventing its complete turnover by CYP enzymes. In a strain expressing HFS (CYP76AH22) residual amounts of ferruginol are present but the concentration ratio of 11-hydroxyferruginol to ferruginol is 18.17, indicating an almost complete conversion of ferruginol to 11-hydroxyferruginol. Interestingly, the strain expressing the triple mutant FS_D301E,N303S,V479F_ has very similar yields and ratios, notably 11-hydroxyferruginol to ferruginol of 18.12, confirming the analysis by GC–MS and LC–MS. In the strain co-expressing the HFS and the C_20_Ox, neither 11-hydroxyferruginol nor any of the intermediates of the C20 oxidation could be detected by NMR, indicating a quantitative conversion to CA by C_20_Ox. Furthermore, the concentration yield was the highest of all products with 8.26±1.64 μmol l^−1^ of CA and a ratio of CA to ferruginol of 21.18. The higher yields of CA could be explained both by the high activity of the C_20_Ox enzymes, but also by the higher hydrophilicity of CA, which is more water soluble than miltiradiene or ferruginol, and is therefore less likely to be trapped in membranes. In the combination of FS with C_20_Ox, which is not a natural combination, as the C_20_Ox comes from rosemary and the ferruginol synthase from *S. miltiorrhiza*, the situation is different as only 1.82±0.75 μmol l^−1^ of PA can be produced, with a ratio of 0.76 to miltiradiene. Interestingly, higher concentrations of pisiferal, the aldehyde intermediate by C_20_ oxidation of ferruginol, are produced (3.03±0.84 μmol l^−1^). This supports a higher specificity of the C_20_Ox for 11-hydroxyferruginol compared with ferruginol. The absence of the hydroxyl group at the C11 position further prevents the efficient conversion of the aldehyde (pisiferal) to the end product, PA. A graphical overview of the results of phenolic diterpene pathway engineering is provided in Fig. 4.

### Phylogeny of CYP76AH and CYP76AK proteins

Using the full-length amino acid sequences, a phylogenetic analysis was applied to the investigated CYP76AH and CYP76AK enzymes (Supplementary Table 7 and Supplementary Fig. 22). The resulting maximum-likelihood tree confirmed that they belong to the CYP76 clan whose members are often involved in the oxidation of terpenes^32^. As expected, they share the highly conserved CYP motifs, that is, the I-helix (AGxDT), K-helix (KETLR), PERF and haem-binding domain (PFGxGRRxCPG) (Supplementary Figs 1 and 2)^25^. HFS and C_20_Ox cluster into subgroups, which form a subfamily with SmCYP76AH1, MgCYP76AH2, MgCYP76AH9 and several CYP76 members from *Coleus forskohlii*, the latter involved in the biosynthesis of forskolin and related compounds^33^. The HFSs (CYP76AH4, 22–24) constitute a clade distinct of the FS (CYP76AH1) reflecting their functional differences. Interestingly, CYP76AH1 only shares 79% sequence identity with CYP76AH24, in contrast to 90–94% between CYP76AH22–24, although both originate from sage species. Therefore, the presence of rosemary and sage sequences in the HFS clade indicates an origin predating the divergence of these species. Whether HFS evolved from FS or the opposite cannot be concluded with the current data set. The sequence similarity within the C_20_Ox cluster ranges from 70 to 77%, whereby CYP76AK8 from *R. officinalis* is more similar to CYP76AK6 from *S. fruticosa* than to its rosemary homologue CYP76AK7. This analysis underscores the importance of the CYP76 clan in the oxidation of diterpenes, particularly in the Lamiaceae family, which is particularly rich in diverse labdanoid diterpene structures.

We note that almost identical genes were identified and characterized from *S. pomifera*, which when expressed in yeast also lead to production of CA^28^. By identifying three amino acid residues in the FS that recapitulate HFS activity and with *in vivo* quantification data, our results provide additional insights into the structure activity relationship of this family of CYP enzymes involved in the oxidation of diterpenes.

In conclusion, the discovery of the missing genes for the biosynthesis of CA makes it possible to engineer yeast, but also other organisms including plants, for the production at high levels of this industrially important compound. It demonstrates the power of enzymatic synthesis of high value products even compared with sophisticated chemical synthesis^34^. The combinatorial approach used for CA biosynthesis in yeast constitutes a versatile and efficient platform for the elucidation and production of other related terpenoids, including the promising anti-cancer tanshinones.

## Methods

### Plant material

*R. officinalis* was obtained from the Conservatoire National des *Plantes à Parfum, Médicinales, Aromatiques et Industrielles* (http://www.cnpmai.net/). *S. fruticosa* plants were kindly provided by the group of Professor Angelos Kanellis^16^. *N. benthamiana*, *R. officinalis* and *S. fruticosa* plants were grown in the greenhouse under long day conditions (16 h light/8 h darkness) with temperatures of 25 °C during day and 20 °C at night, with 53% humidity. Leaf material for gene isolation and chromatographic analysis was collected in the vegetative phase.

### Chemicals

Authentic standards of CA, CO and PA were purchased from Santa Cruz Biotechnology Inc., Sigma Aldrich and TCI Europe, respectively.

### Tissue collection and RNA preparation for quantitative RT–PCR

Leaves from *R. officinalis* and *S. fruticosa* from Kavoussi, Crete, were collected in two developmental stages, young (1–2 cm) and aged (3–4 cm) leaves. Trichomes from leaves were isolated using dry ice^35^. An additional brush-abrasion step was used to remove any leftover trichomes from leaf tissue. The plant material was homogenized and RNA isolation was performed using Spectrum Plant Total RNA Kit (Sigma-Aldrich) with additional ethanol washing steps. The complementary DNA was generated from 1 μg total RNA using Superscript III kit (Invitrogen) according to manufacturers' specifications and with 1 μg random primers (Invitrogen).

### Quantitative real-time PCR analysis

The PCR mix was prepared using KAPA SYBR FAST qPCR kit and quantitative real-time PCR analysis was performed in triplicates using the Applied Biosystems 7500 Real-Time PCR System (Invitrogen) and the following PCR conditions: 95 °C for 2 min; 6 cycles of 95 °C for 35 s, 64 °C for 30 s, 72 °C for 30 s; 35 cycles of 95 °C for 20 s, 62 °C for 20 s, 72 °C for 15 s; 72 °C for 10 min; plate read at 76 °C. To receive a melting curve from 70 °C to 95 °C, data points were collected every 0.1 °C with a 10 s hold between them. The data were evaluated using the delta Ct method^36^ with the elongation factor 4a as reference gene.

### Construction of golden gate compatible yeast expression MoClo vectors

Construction of Golden Gate compatible yeast expression vectors of level 1 and M was carried out as for the MoClo system^37^,^38^. Their backbones were equipped with origins of replication for *Escherichia coli* (ColE1) and *Saccharomyces cerevisiae* (2 μm ori) from pYES2 (Invitrogen), and in addition contained *E. coli* antibiotic selection markers for replication (carbenicillin in level 1 vector and spectinomycin in level M vector). To select for insertion into the vectors, a *LacZ* gene flanked by BsaI (in level 1 vector) or BpiI restriction sites (in level M vector) were introduced into the backbones. To allow assembling of up to six level 1 transcription units in a defined order in level M vectors, level 1 vectors contained BpiI restriction sites, which produced specific 4 bp overhangs dependent on the position on restriction^37^. The last positioned insert was connected to the vector M backbone by sequence specific linkers. An *URA3* gene completed the backbone for selection of yeast transformants.

### Designing synthetic galactose-inducible promoters

Degenerated sequences of synthetic galactose-inducible promoters (Supplementary Table 2) were created by primer extension PCR (primers are given in Supplementary Table 1). They contained a degenerated consensus sequence of 147 bp of pGAL1 and pGAL2 as core promoter and four GAL4-binding domains (three separated by 2 bp, the fourth with 20 bp distance)^39^.

### Isolation and cloning of genes

Plasmids containing *GGPPS*, *CPS*, *MS* and *ATR1* were kindly provided by colleagues within the institute^12^,^40^,^41^. The cDNA sequences of CYP76AH1 (accession number JX422213.1) and CYP76AH4 were obtained from the literature^14^,^15^. Total RNA from *R. officinalis* leaves was isolated and transcribed to cDNA using Spectrum Plant Total RNA Kit (Sigma-Aldrich) and RevertAid Premium First Strand cDNA Synthesis Kit (Fermentas), respectively. Full-length coding sequences for CYP76AK6-8 were PCR amplified from *R. officinalis* or *S. fruticosa* cDNAs using specific primers (Supplementary Table 1) and AccuPrime High Fidelity Taq Polymerase (Invitrogen). The amplified fragments were gel-purified and subcloned into pENTR-D-TOPO vector (Invitrogen). The cDNAs, together with p35S and tOCS, were transferred into pEarleyGate100 binary vector^42^ using Gateway LR Clonase II Enzyme mix (Invitrogen). Constructs were used for further cloning or for transient expression in *N. benthamiana*.

To produce plasmids for yeast engineering, Golden Gate compatible yeast expression vectors were used. *CYP76AH22*, *CYP76AH23*, *CYP76AH24*, *CYP76AH4*, *CYP76AK6*, *CYP76AK7* and *CYP76AK8* were PCR amplified in fragments, to remove internal BpiI and BsaI restriction sites by introducing single point silent mutations. The coding sequences were amplified from either plasmids or cDNA with KOD Hot Start DNA Polymerase (Merck) and gene-specific primers (Supplementary Table 1). PCR products were then purified using QIAquick PCR Purification Kit (Qiagen) and subsequently sequenced. As the amino terminus of *CYP76AH4* could not be amplified from cDNA, a mixed sequence out of a *CYP76AH22*-specific N terminus (253 amino acids) and a *CYP76AH4*-specific carboxy terminus (243 amino acids) was used for further analysis (Supplementary Fig. 1). *CYP76AH1* was synthesized by Eurofins Genomics GmbH as a codon-optimized sequence for yeast expression. Cloning into Golden Gate entry vector pL0-SC and yeast expression vector 1 was performed as previously described^43^. In further cloning steps, each gene was fused to a synthetic galactose-inducible promoter and a terminator (Supplementary Table 2) in a yeast level 1 expression vector using BsaI (Fermentas) and T4-Ligase in a 50 cycle restriction–ligation reaction. Different gene combinations were finally assembled into one yeast expression level M vector by a 50 cycle restriction–ligation reaction with BpiI and T4-Ligase.

### Phylogenetic analysis

Amino acid sequences (Supplementary Table 7) were aligned and trimmed using MAFFT version 7 with default settings (http://mafft.cbrc.jp/alignment/server/) and TrimAl version 1.3 with the method Authomated1 (http://phylemon2.bioinfo.cipf.es/utilities.html), respectively. The phylogenetic tree was generated using Mega6 (ref. 44) with the maximum likelihood method based on the JTT/+G model (five categories)^45^ and a bootstrap of 1,000 replicates.

### Site-directed mutagenesis

Site-directed mutagenesis of Golden Gate entry vector pL0-SC containing cDNAs for CYP76AH22 or CYP76AH1 was performed using the QuikChange II Site-Directed Mutagenesis Kit (Agilent Technologies). Used primers are given in Supplementary Table 1. One microlitre of the reaction mixture was transformed into *E. coli* cells strain DH10B and plated onto agar plates containing appropriate antibiotics. Mutagenized plasmids were subsequently cloned as described above in yeast expression vectors.

### Yeast microsome isolation

Microsomal preparation was done according to the literature^40^ with the following adaptations. The plasmids with the P450 encoding gene and *ATR1* were transformed in yeast strain INVSc1 as described above. A single positive colony was used to inoculate 5 ml of Ura-medium with 2% glucose and grown for 24 h at 30 °C with shaking. The culture was then used to inoculate 100 ml of fresh Ura-medium with 2% glucose in a 500 ml flask, which was then further agitated at 30 °C for 24 h. The cells were then collected by centrifugation, resuspended in 100 ml yeast extract-peptone-dextrose (YPD) medium with 2% galactose to induce expression and grown with shaking for 24 h at 30 °C. All steps were then carried out at 4 °C. The cells were recovered by centrifugation and resuspended in 30 ml of pre-chilled TEK buffer (50 mM Tris-HCl pH 7.5, 1 mM EDTA, 100 mM KCL), centrifuged again and resuspended in 2 ml TES buffer (50 mM Tris-HCl pH 7.5, 600 mM sorbitol, 10 g l^−1^ BSA, 1.5 mM β-mercaptoethanol) and transferred to a 50 ml tube. Acid-washed autoclaved 450–600 μm diameter glass beads were then added until they reached the surface of the cell suspension. The suspension was shaken vigorously by hand for 1 min and returned to ice for 1 min. This was repeated four times. To wash the glass beads, 5 ml TES buffer was added, after careful inversion the supernatant was collected and transferred to a new tube. The procedure was repeated three times. The tube was then centrifuged at 7,500 *g* for 10 min, the supernatant transferred to ultracentrifugation tubes, which were centrifuged for 2 h at 100 000 *g*. The supernatant was discarded and gently washed successively with 5 ml TES and 2.5 ml TEG buffer (50 mM Tris-HCl pH 7.5, 1 mM EDTA and 30% glycerol). The pellet was then collected with a spatula to a Potter homogenizer resuspended in 2 ml TEG buffer and carefully homogenized. One hundred microlitre aliquots were transferred to 1.5 ml microtubes and stored at −80 °C until used.

### *In vitro* CYP assays

In a 2.0 ml microtube, in a total volume of 600 μl, 40 μl of the microsome preparation were mixed with 100 μM substrate, 1 mM NADPH, 50 mM sodium phosphate pH and incubated at 30 °C for 2 h with gentle shaking. The reaction was then extracted with 1 ml hexane under strong agitation (vortex). After centrifugation, the organic phase was transferred to fresh tubes, dried under a N_2_ stream and resuspended in 50 μl hexane for GC–MS analysis or 150 μl methanol for LC–MS analysis.

### Transient expression in *N. benthamiana*

The cDNAs for CPS, MS, ATR1 and CYP76AK7 were cloned into T-DNA vectors (binary vector pL1F-1) under the control of the 35S promoter and flanked by the Ocs terminator^46^. The resulting T-DNA plasmids were transformed into *Agrobacterium tumefaciens* strain GV3101::pMP90 (resistances to gentamycin and rifampicin) and streaked onto LB agar plates with appropriate antibiotics. Positively transformed colonies were inoculated into LB medium with selective antibiotics and grown for 18 h at 28 °C. To co-infiltrate several genes, appropriate volumes required to reach a final OD_600_ of 0.5 each were combined, centrifuged and resuspended in one-fourth volume fresh LB medium, one-fourth volume sterile water, half volume twofold concentrated infiltration medium (0.3 M sucrose, 20 mM glucose, 8.6 g l^−1^ Murashige and Skoog medium (Duchefa Biochemie), adjusted to pH 5.6) and 20 μM acetosyringone (diluted in dimethyl sulfoxide). The suspension was infiltrated into the abaxial leaf side of 4-week-old *N. benthamiana* plants using a syringe without needle. After treatment, the plants were cultivated under regular growth conditions for 5 days.

### Homology modelling

Protein homology modelling of CYP76AH1, CYP76AH4, CYP76AH22, CYP76AH23 and CYP76AH24 was performed with YASARA^47^. After search for templates in the protein database^48^ for each protein, 100 models were created based on alternative sequence alignments including secondary structure predictions and comparisons with ten found appropriate X-ray templates of different CYPs^49^. The best suited template was the X-ray structure of 2HI4 (refs 50, 51). Models based on some other templates such as 2NNJ^52^, 3T3Q^53^, 2PG5 (ref. 54) and 4NKY^55^ are almost identical, except in some loop conformations. The substrates were docked using the molecular modelling environment programme MOE 2014.09 (https://www.chemcomp.com/). All models with docked substrates in appropriate position for an oxidation reaction in vicinity to the iron ion complexed by haem were refined with a 20 cycle simulated annealing procedure (md-refine.mcr) in YASARA. For creating the figures, the reactive oxygen atom bound to the iron ion was added manually in MOE corresponding to the proposed catalytic mechanism^50^. The quality of the resulting homology models were evaluated using PROCHECK^56^ and ProSA^57^,^58^. Despite relatively low sequence identities of the enzymes with their template, all models were of excellent quality with >92% of the residues being in the most favoured region of the Ramachandran plot. The ProSA energy graphs were all in negative range and the calculated *z*-scores (for CYP76AH1=−10.84 with 478 amino acid residues in the model and for CYP76AH22=−11.04 with 481 amino acid residues) are in the range of natively folded proteins.

### Production and quantification of diterpenes in yeast

Yeast expression vectors were transformed into *S. cerevisiae* strain INV*Sc*1 (genotype: *MATa his 3D1 leu2 trp1-289 ura3-52*; Thermo Fisher Scientific) and plated out onto uracil-free selection medium (1 g l^−1^ Yeast Synthetic Drop-out Medium Supplements without uracil (Sigma-Aldrich), 6.7 g l^−1^ Yeast Nitrogen Base With Amino Acids (Sigma-Aldrich) and 20 g l^−1^ Micro Agar (Duchefa Biochemie)). Positively transformed colonies were inoculated into 5 ml YPD medium (20 g l^−1^ tryptone and 10 g l^−1^ yeast extract) containing 2% of glucose and grown for 24 h with shaking at 30 °C. To induce protein expression, the cell pellet was resuspended in fresh YPD medium containing 2% galactose. After another 24 h of cultivation, whole cultures were extracted with 2 ml *n*-hexane. Quantification of shake-flask expression was done according to the literature with the following adaptions^15^. Two single yeast colonies carrying the plasmids were inoculated into 5 ml selection medium (1 g l^−1^ Yeast Synthetic Drop-out Medium Supplements without uracil (Sigma-Aldrich) and 6.7 g l^−1^ Yeast Nitrogen Base With Amino Acids (Sigma-Aldrich)) each. After cultivation for 24 h, they were transferred to 50 ml selection medium containing 2% glucose in a 250 ml flask and grown for another 24 h. Expression was induced the next day by resuspending the cell pellet in 50 ml YPD medium containing 2% galactose. After cultivation for 24 h the optical density (OD_600_) was determined and a volume with comparable cell number between all samples was used to extract the formed products with 45 ml hexane. An aliquot was used for analysis by GC–MS and LC–MS as described below. The extracts were then evaporated to complete dryness and dissolved in 800 μl C_6_D_6_ containing hexamethyldisiloxane (HMDS) at a concentration of 0.0235, mmol l^−1^ as internal standard for chemical shift referencing (δ^1^H HMDS: 0.112 p.p.m.) as well as quantification.^1^H NMR spectra were recorded on a Varian/Agilent VNMRS 600 NMR spectrometer operating at a proton NMR frequency of 599.83 MHz using a 5 mm inverse detection cryoprobe with the following parameters: pulse width=6.25 μs (90° flip angle), relaxation delay=27.3 s, acquisition time=2.7 s, number of transients=40 and digital resolution 0.367 Hz per point. An exponential multiplication of the free induction decays were performed using a line broadening factor lb=0.4 Hz. A zero filling by a factor of 2 was used before Fourier transformation. All spectra were manually phase corrected and baseline corrected. For the quantification of metabolites, the peak area of selected proton signals belonging to the target compounds and the peak area of the internal standard (HMDS) were integrated manually. Peak assignments were done according to chemical shifts of reference compounds based on commercial standards or characterization of metabolites performed in this work.

### GC–MS analysis

Plant surface extracts (*R. officinalis*, *S. fruticosa* and infiltrated *N. benthamiana*) were prepared by shaking three young leaves (*R. officinalis* and *S. fruticosa*) or ten leaf discs (7 mm diameter in case of *N. benthamiana*) with 1 ml *n*-hexane for 2 min at room temperature. The extract was evaporated to complete dryness and resuspended in 200 μl *n*-hexane. The analysis of yeast and plant extracts were carried out using a Trace GC Ultra gas chromatograph (Thermo Scientific) coupled to ATAS Optic 3 injector and an ISQ single quadrupole mass spectrometer (Thermo Scientific) with electron impact ionization. Chromatographic separation was performed on a ZB-5ms capillary column (30 m × 0.32 mm, Phenomenex) using splitless injection and an injection volume of 1 μl. The injection temperature rose from 60 °C to 250 °C with 10 °C s^−1^ and the flow rate of helium was 1 ml min^−1^. The GC oven temperature ramp was as follows: 50 °C for 1 min, 50 to 300 °C with 7 °C min^−1^, 300–330 °C with 20 °C min^−1^ and 330 °C for 5 min. Mass spectrometry was performed at 70 eV, in a full scan mode with *m/z* from 50 to 450. Data analysis was done with the device specific software Xcalibur (Thermo Scientific).

### RP–UPLC–ESI–MS/MS analysis (LC–MS)

Hexane leaf surface extracts of *R. officinalis* and *S. fruticosa* leaves were completely evaporated under a nitrogen stream, dissolved in 150 μl methanol and subjected to reversed phase–ultra-performance LC–electrospray ionization (ESI)–MS/MS analysis. Metabolites were separated using a Nucleoshell RP18 column (2 × 150 mm, particle size 2.7 μm, Macherey-Nagel) and a ACQUITY UPLC System (Waters), including an ACQUITY Binary Solvent Manager and an ACQUITY Sample Manager (10 μl sample loop, partial loop injection mode, 5 μl injection volume). For elution, aqueous 0.3 mmol l^−1^ NH_4_HCOO (adjusted to pH 3.5 with formic acid) (A) and acetonitrile (B) were used. The elution conditions were as follows: isocratic from 0 to 2 min at 5% eluent B, from 2 to 19 min linear from 5 to 95%, from 19 to 22 min isocratically at 95%, from 22 to 22.01 min linear from 95 to 5% and from 22.01 to 24 min isocratically at 5% eluent B. During the separation, the flow rate was set to 400 μl min^−1^ and the column temperature was maintained at 40 °C. A TripleToF 5600-1 mass spectrometer (AB Sciex) was used to detect the metabolites, which was equipped with an ESI-Duo-TurboIon-Spray interface (it operated in negative ion mode) and was controlled by Analyst 1.6 TF software (AB Sciex). The LC–ESI source operation parameters were as follows: ion spray voltage: −4,500 V, nebulizing gas: 60 p.s.i., source temperature: 600 °C, drying gas: 70 p.s.i., curtain gas: 35 p.s.i. Data acquisition was performed in the MS^1^-ToF mode^59^, scanned from 250 to 500 Da with an accumulation time of 50 ms, and the MS^2^-SWATH mode, divided into 5 Da segments of 20 ms accumulation time. Fifty-six separate scan experiments were carried out covering the mass range from 65 to 500 Da. In this process, a declustering potential of 35 V (−35 V) was applied and collision energies were set to 55 V with a collision energy spread of ±45 V.

### Isolation of new PDs and structure confirmation by NMR

For isolation of 11-hydroxyferruginol, a single yeast colony expressing GGPPS, CPS, MS, ATR1 and CYP76AH22 was inoculated into 5 ml YPD medium containing 2% glucose and grown at 28 °C with shaking. After 24 h, the suspension was transferred into 400 ml culture medium for shake-flask expression and grown for another 24 h. The expression was induced by resuspending the cell pellet in 400 ml YPD with 2% galactose and grown as mentioned. The diterpenes were extracted from the yeast culture by adding 300 ml *n*-hexane and shaking thoroughly. Miltiradien-20-al was isolated from 84 g infiltrated leaf material of *N. benthamiana* expressing GGPPS, CPS, MS and the C_20_Ox CYP76AK6, which were homogenized and extracted with 800 ml *n*-hexane. The extract was separated from leaf material by centrifugation, evaporated and dissolved in 50 ml *n*-hexane. To initially separate miltiradien-20-al from other leaf compounds, the extract was applied to a SiOH column (25 g). Elution steps of 99:1, 98:2, 97:3 and 96:4 *n*-hexane:ethyl acetate were collected and the last fraction containing miltiradien-20-al was used for preparative high performance LC. Carnosaldehyde was extracted twice from 100 young leaves of *R. officinalis* with 80 ml *n*-hexane each time. The combined extracts were evaporated, dissolved in 40 ml 95:5 *n*-hexane:ethyl acetate and applied to a SiOH column (15 g), which was equilibrate with 95:5 *n*-hexane:ethyl acetate. Carnosaldehyde eluted in the 94:6 *n*-hexane:ethyl acetate fraction which was used for further purification.

All enriched fractions were applied to a rotary evaporator and the dried products were resuspended in 1 ml of methanol. Semi-preparative separation of 11-hydroxyferruginol and carnosaldehyde was done by LC-UV (Waters 2695 with 2996 diode array detector, Waters GmbH) equipped with a fraction collector (Waters, FC III). Between the column and fraction collector, a capillary splitter (1:100) was installed which allowed ultraviolet–visible analysis at 254 nm and fraction collection. Using an XTerra Prep C18 column (7.8 mm × 150 mm × 5 μm, Waters GmbH) and a flow rate of 6 ml min^−1^, the same LC gradient was applied as for reverse phase–ultra-performance LC–ESI–MS/MS analysis. One hundred microlitres of each enriched fraction were injected repeatedly (four times) and a fraction eluting between 17 and 18 min (11-hydroxyferruginol) or between 16.1 and 16.5 min (carnosaldehyde) was repeatedly collected. The fractions were combined, dried down in a nitrogen stream and resuspended in deuterated solvents for NMR analysis. ^1^H and two-dimensional spectra were recorded on an Agilent VNMRS 600 NMR spectrometer operating at 599.832 MHz using a 5 mm inverse detection cryoprobe. Chemical shifts were referenced to internal tetramethylsilane (*δ*=0 p.p.m., ^1^H) or internal C_6_D_11_H (*δ*=1.38 p.p.m., ^1^H) and CDCl_3_ (δ=77.0 p.p.m., ^13^C), or C_6_D_12_ (δ=26.4 p.p.m., ^13^C) respectively.

### Data availability

Data supporting the findings of this study are available within the article and its Supplementary Information files, and from the corresponding author upon reasonable request. The sequences of the CYP76AK6, CYP76AK7 and CYP76AK8 enzymes are available under GenBank accessions KX431218, KX431219 and KX431220, respectively.

## Additional information

**How to cite this article:** Scheler, U. *et al*. Elucidation of the biosynthesis of carnosic acid and its reconstitution in yeast. *Nat. Commun.* **7**, 12942 doi: 10.1038/ncomms12942 (2016).

## Supplementary Material

Supplementary Information Supplementary Figures 1-22, Supplementary Tables 1-7 and Supplementary References.

## Figures and Tables

**Figure 1:**
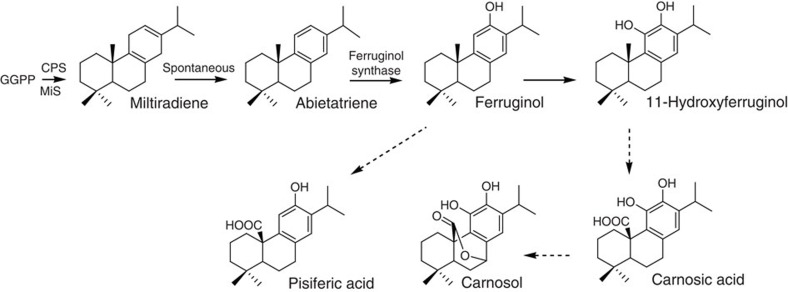
Biosynthesis of PDs in *Rosmarinus officinalis* and sage species. State of the knowledge on the biosynthetic pathway from GGPP to CA, CO and PA before this study. The terpene synthases CPS and MiS cyclize GGPP to miltiradiene, which spontaneously oxidizes to abietatriene. Ferruginol synthases oxidize abietatriene to ferruginol. A further intermediate is presumed to be 11-hydroxyferruginol. Oxidations at the C20 position should lead to pisiferic acid (PA) from ferruginol and to carnosic acid (CA) from 11-hydroxyferruginol. Further oxidation of CA at the C7 position results in carnosol.

**Table 1:** Quantification by NMR of products formed in engineered yeast strains.

**Expressed enzymes**	**Product**	**Concentration (μmol** **l**^**−1**^**)**	**Concentration (mg** **l**^**−1**^**)**	**Ratio product/miltiradiene**	**Ratio product/ferruginol**
Empty vector control	ND	−	−		
GGPPS:CPS:MiS	Miltiradiene	5.86±1.52	1.59±0.41	1	
GGPPS:CPS:MiS:ATR1:	Miltiradiene	0.93±0.18	0.25±0.05	1	0.15
CYP76AH1	Ferruginol	6.37±0.55	1.82±0.16	6.85	1
GGPPS:CPS:MiS:ATR1:	Miltiradiene	0.86±0.22	0.23±0.06	1	3.74
CYP76AH22	Ferruginol	0.23±0.05	0.07±0.01	0.27	1
	11-hydroxyferruginol	4.18±0.48	1.26±0.14	4.86	18.17
GGPPS:CPS:MiS:ATR1:	Miltiradiene	1.15±0.11	0.31±0.03	1	2.80
CYP76AH1_D301E,N303S,V479F_	Ferruginol	0.41±0.09	0.12±0.03	0.35	1
	11-hydroxyferruginol	7.43±1.20	2.25±0.36	6.46	18.12
GGPPS:CPS:MiS:ATR1:	Miltiradiene	3.72±0.25	1.01±0.07	1	0.558
CYP76AH22_E301D,S303N,F478V_	Ferruginol	6.78±0.20	1.94±0.06	1.82	1
GGPPS:CPS:MiS:ATR1:	Miltiradiene	0.73±0.14	0.20±0.04	1	1.87
CYP76AH22:CYP76AK8	Ferruginol	0.39±0.09	0.11±0.03	0.53	1
	Carnosic acid	8.26±1.64	2.74±0.55	11.31	21.18
GGPPS:CPS:MiS:ATR1:	Miltiradiene	2.41±0.25	0.66±0.07	1	3.60
CYP76AH1:CYP76AK8	Ferruginol	0.67±0.10	0.19±0.03	0.28	1
	Pisiferal	3.03±0.84	0.91±0.25	1.25	4.52
	Pisiferic acid	1.82±0.75	0.57±0.24	0.76	2.72

CPS, copalyl diphosphate synthase; GGPPS, geranylgeranyl diphosphate synthase; MS, miltiradiene synthase; ND, not detected.

The concentrations of products, which could be detected with a signal sufficient for quantification, are given with their s.d. (*n*=3). The concentrations are normalized to the cell density as measured by OD_600_. The ratios product/miltiradiene or product/ferruginol were calculated with the molar concentrations.
