# Supplementary material for: Elucidation of the biosynthesis of carnosic acid and its reconstitution in yeast
Source: Nat Commun. 2016 Oct 5;7:12942. doi: 10.1038/ncomms12942 (PMC5059481; doi:10.1038/ncomms12942)
Supplement: Supplementary Information — Supplementary Figures 1-22, Supplementary Tables 1-7 and Supplementary References. [file ncomms12942-s1.pdf]

Supplementary Figures

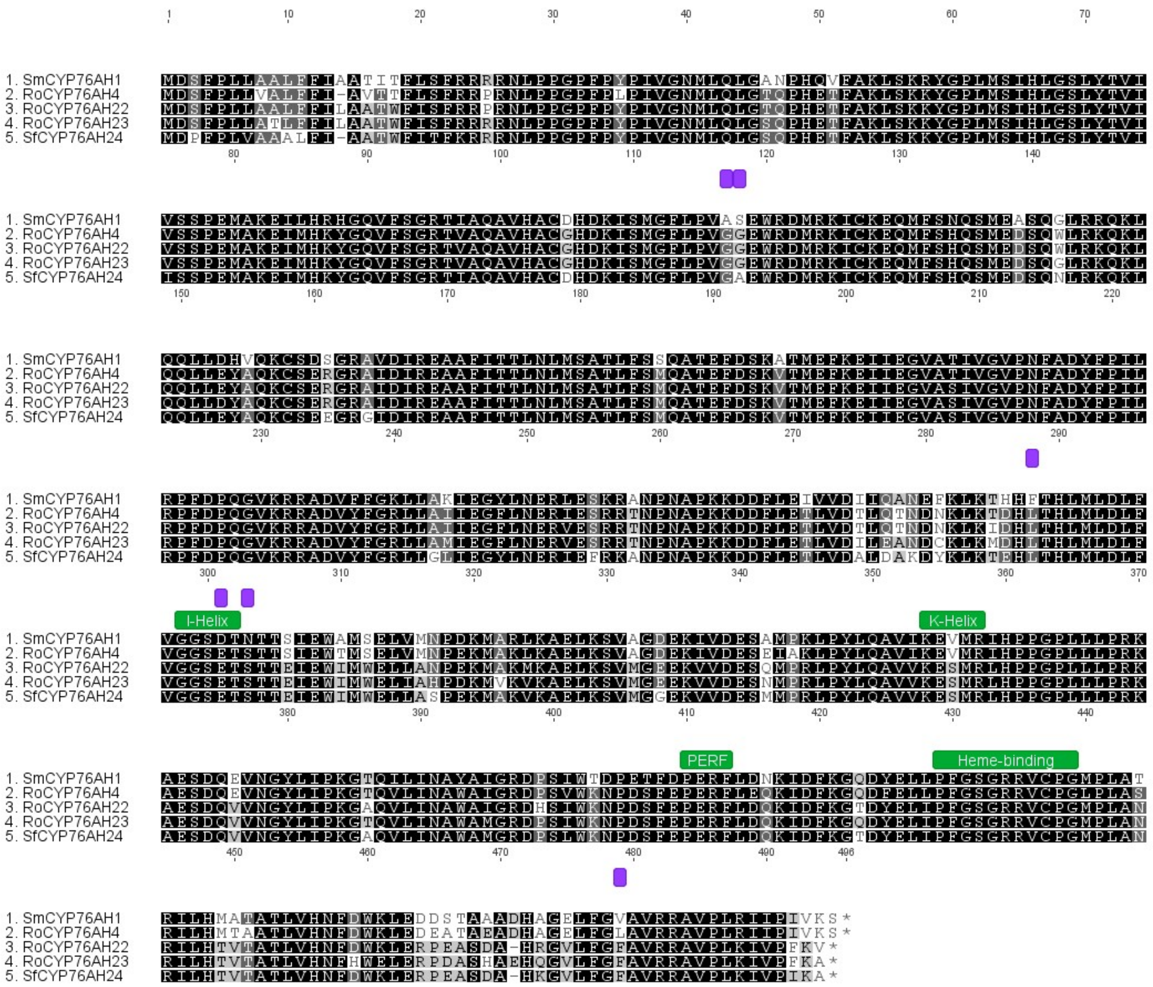

Supplementary Figure 1: Multiple amino acid sequence alignment of the FS and HFSs. Protein sequences of CYP76AH1 (*S. miltiorrhiza*), CYP76AH24 (SfFS) (*S. fruticosa*), CYP76AH4 and CYP76AH22-23 (RoFS1 and RoFS2) (*R. officinalis*) are given. Conserved CYP domains (green) and amino acids which were mutagenized (purple) are highlighted.

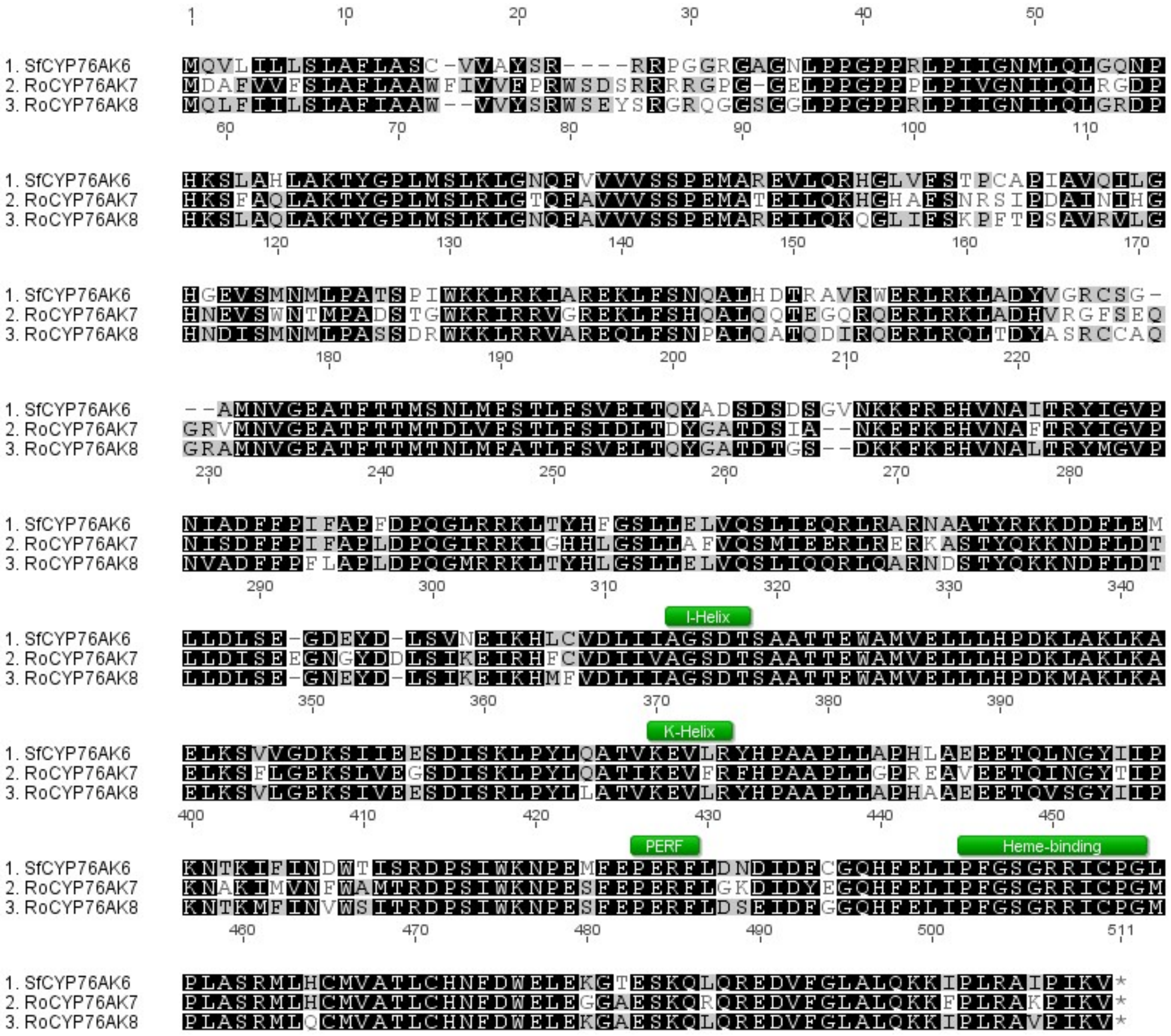

Supplementary Figure 2: Multiple amino acid sequence alignment of the C_{20}Oxs.

CYP76AK6 (*S. fruticosa*) and CYP76AK7-8 (*R. officinalis*) are shown with highlighted conserved CYP domains (green).

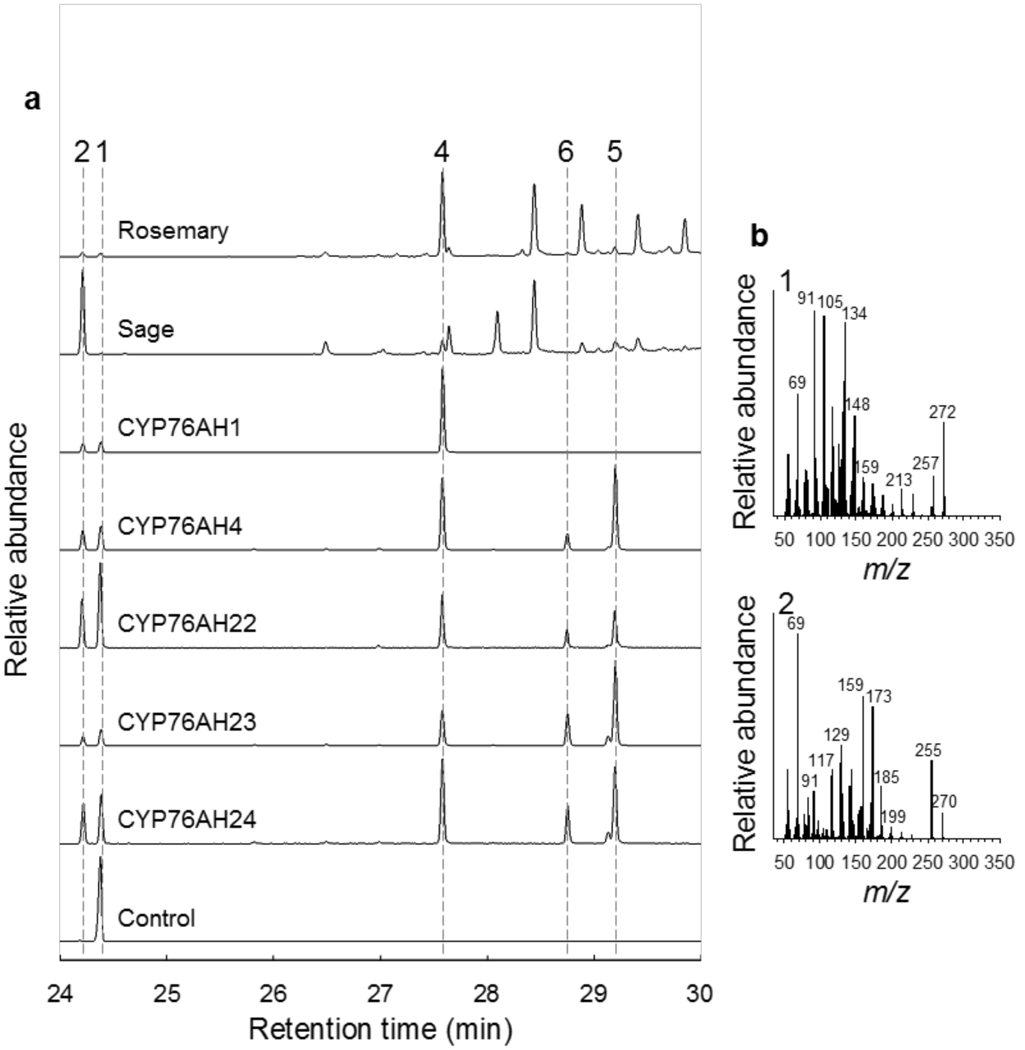

Supplementary Figure 3: GC-MS analysis of yeast expressing FS and HFS. (**a**) *R. officinalis* and *S. fruticosa* leaf surface extracts, and extracts of yeast strains co-expressing GGPPS, CPS, MiS, ATR1 and indicated CYPs (selected *m/z* signals: 270, 272, 286, 300, 302). Miltiradiene (**1**), abietatriene (**2**), ferruginol (**4**), 11-hydroxyferruginol (**5**) and hydroxyferruginol quinone (**6**). (**b**) EI mass spectra of abietatriene (**1**) and miltiradiene (**2**).

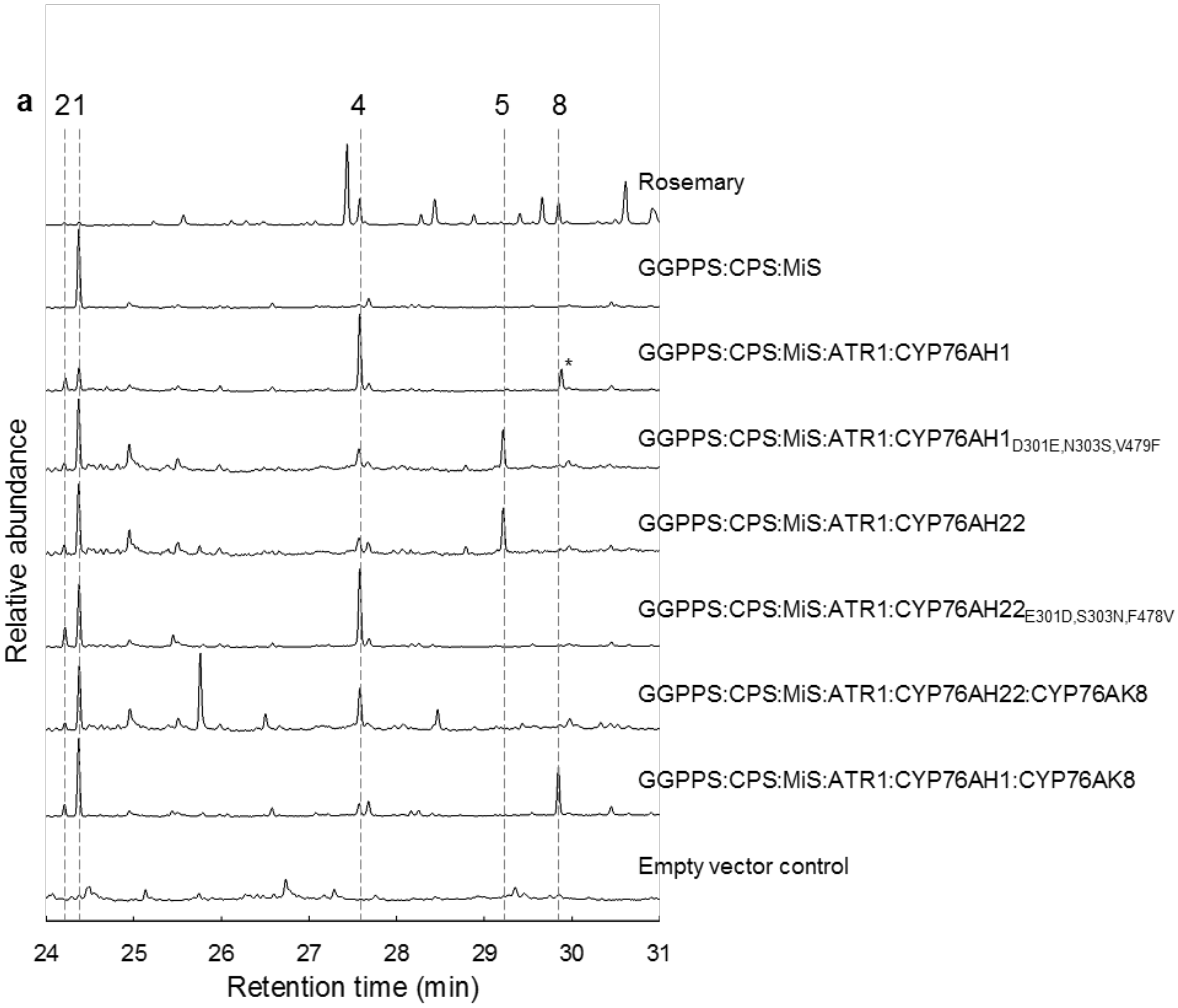
a
2
1
4
5
8
Rosemary
GGPPS:CPS:MiS
GGPPS:CPS:MiS:ATR1:CYP76AH1
*
GGPPS:CPS:MiS:ATR1:CYP76AH1D301E,N303S,V479F
GGPPS:CPS:MiS:ATR1:CYP76AH22
GGPPS:CPS:MiS:ATR1:CYP76AH22E301D,S303N,F478V
GGPPS:CPS:MiS:ATR1:CYP76AH22:CYP76AK8
GGPPS:CPS:MiS:ATR1:CYP76AH1:CYP76AK8
Empty vector control
Relative abundance
24
25
26
27
28
29
30
31
Retention time (min)

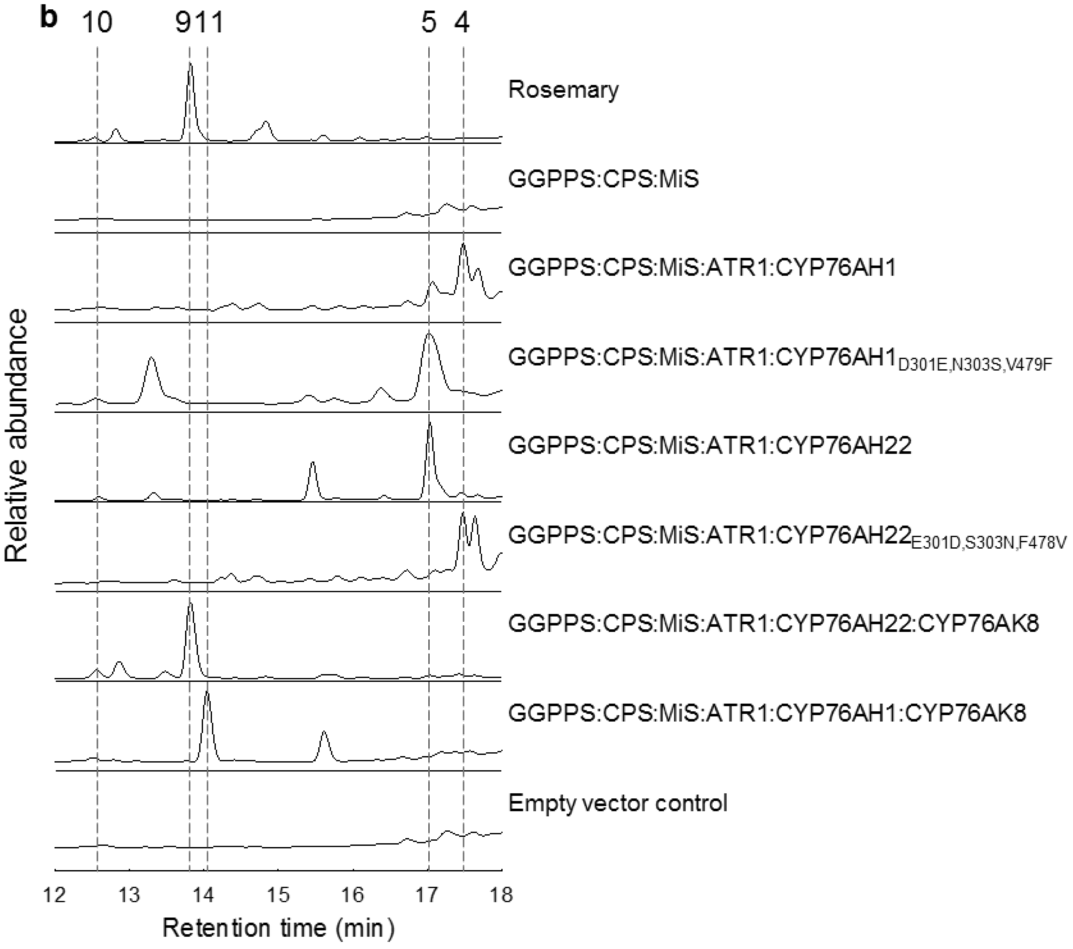

Supplementary Figure 4: Total ion chromatograms of engineered yeast expressing the indicated enzymes and rosemary leaf surface extracts. (**a**) GC-MS analysis. Miltiradiene (**1**), abietatriene (**2**), ferruginol (**4**), 11-hydroxyferruginol (**5**) and pisiferal (**8**). (**b**) LC-MS analysis. CA (**9**), CO (**10**) and PA (**11**). *Originates from yeast.

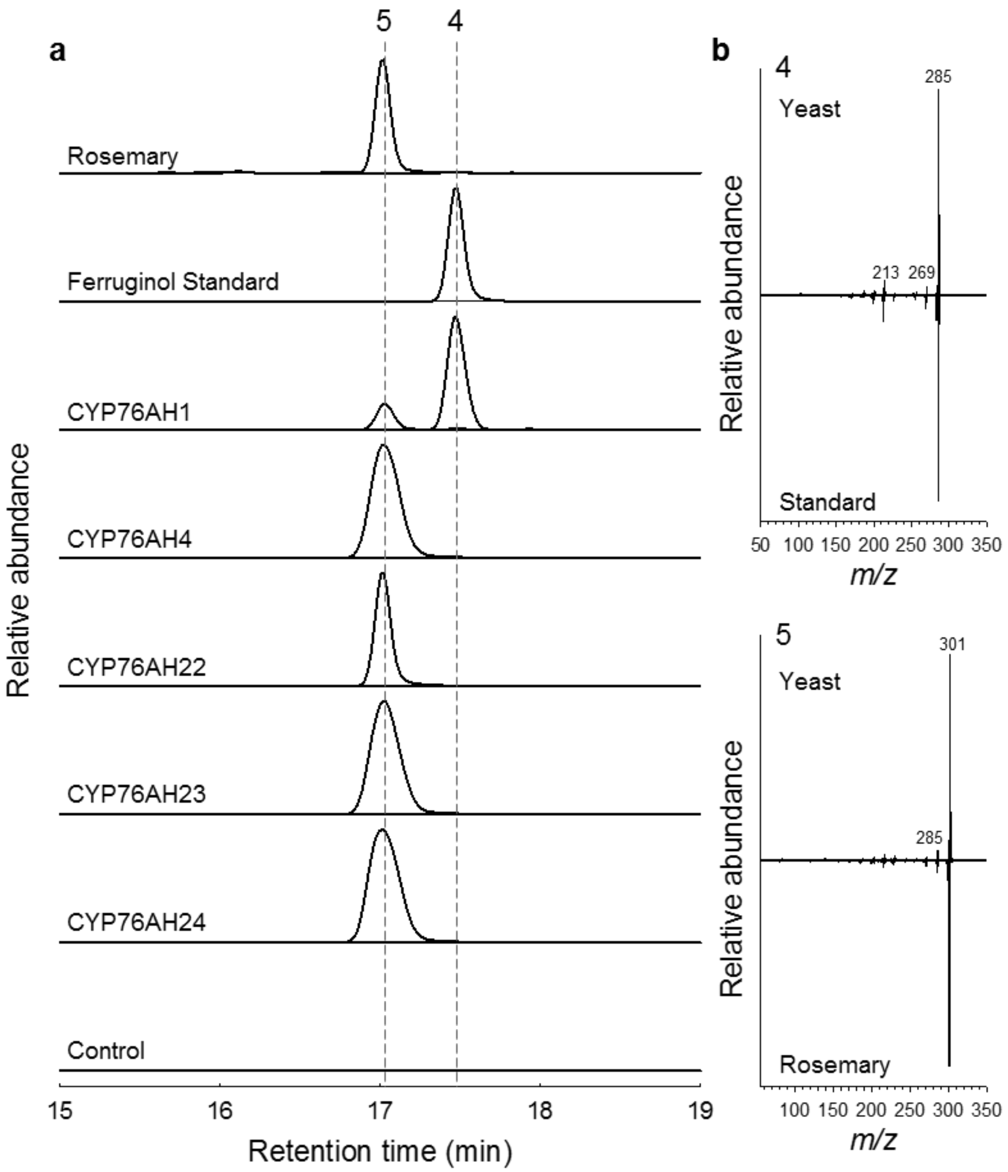

Supplementary Figure 5: LC-MS analysis of yeast expressing FS and HFS. (**a**) Total ion chromatogram of leaf surface extracts from rosemary and sage, and of extracts from yeast co-expressing GGPPS, CPS, MiS, ATR1 and indicated CYPs. Ferruginol (**4**) and 11-hydroxyferruginol (**5**). (selected *m/z* signals: 285.221, 301.217) (**b**) ESI mass spectra of ferruginol (**4**) 11-hydroxyferruginol (**5**) from yeast and rosemary.

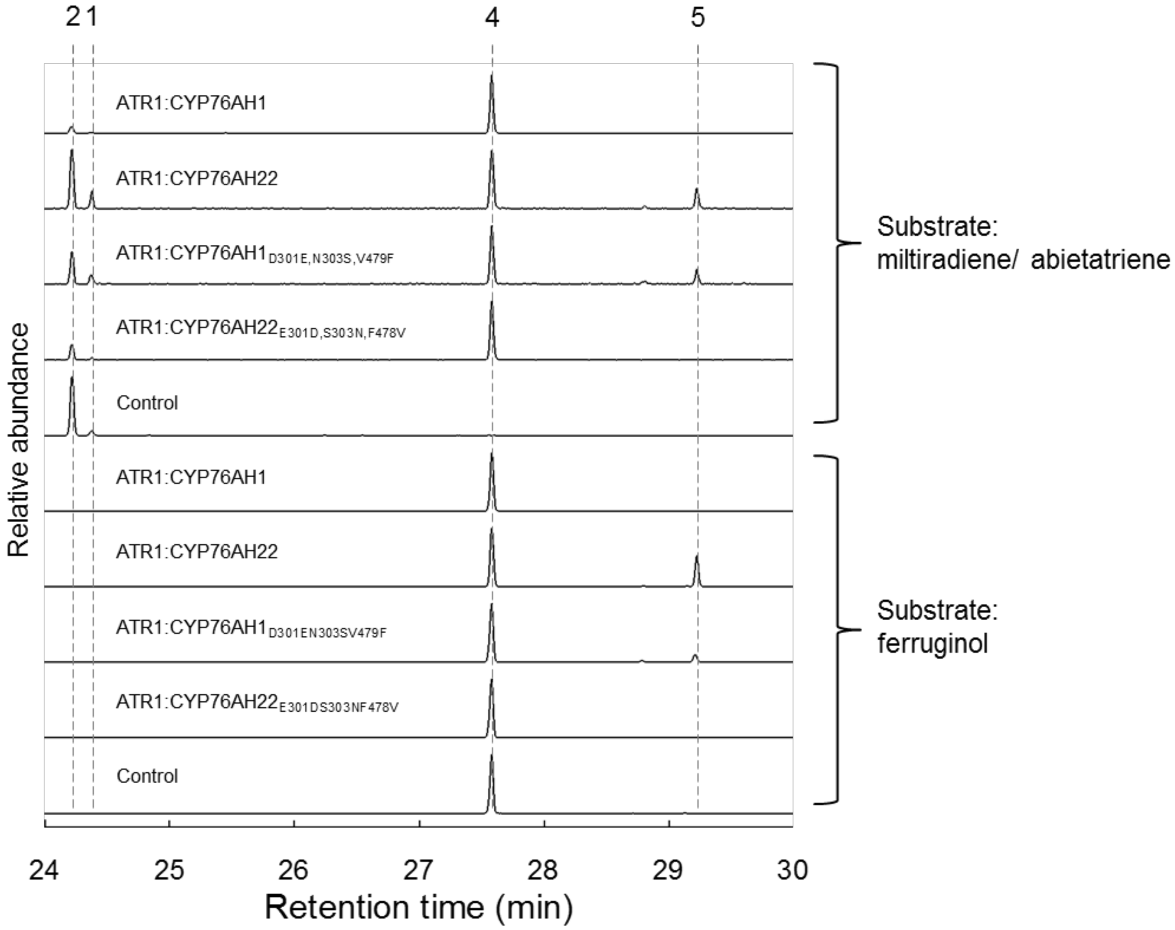

Supplementary Figure 6: GC-MS analysis (selected *m/z* signals: 270, 272, 286, 302) of *in vitro* enzyme assays from microsomal preparations. Indicated enzymes were incubated with the given substrates. Miltiradiene (**1**), abietatriene (**2**), ferruginol (**4**) and 11-hydroxyferruginol (**5**).

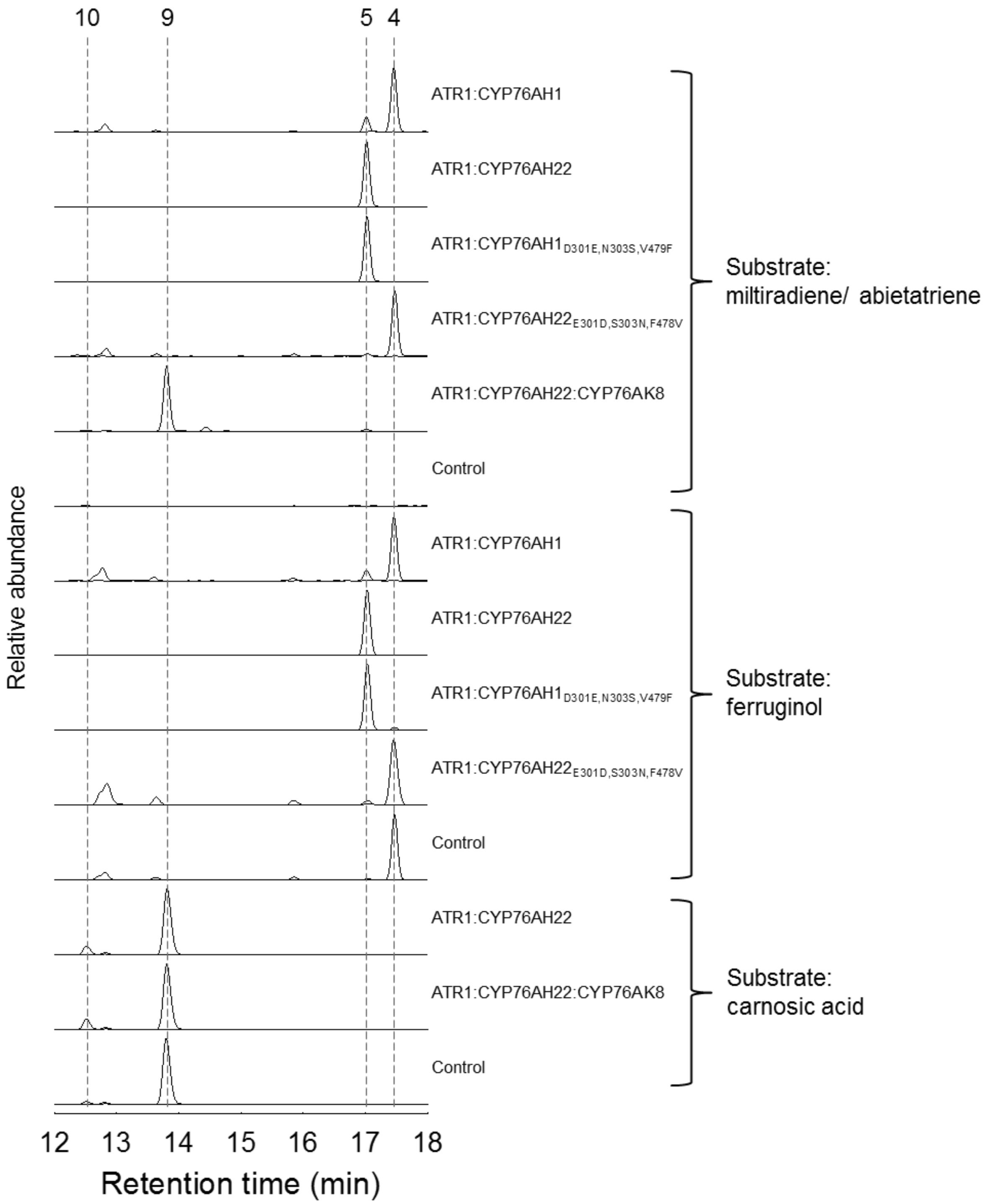

10
9
5
4
ATR1:CYP76AH1
ATR1:CYP76AH22
ATR1:CYP76AH1 D301E,N303S,V479F
ATR1:CYP76AH22 E301D,S303N,F478V
ATR1:CYP76AH22:CYP76AK8
Control
Substrate: miltiradiene/ abietatriene
ATR1:CYP76AH1
ATR1:CYP76AH22
ATR1:CYP76AH1 D301E,N303S,V479F
ATR1:CYP76AH22 E301D,S303N,F478V
Control
Substrate: ferruginol
ATR1:CYP76AH22
ATR1:CYP76AH22:CYP76AK8
Control
Substrate: carnosic acid
Relative abundance
12
13
14
15
16
17
18
Retention time (min)

Supplementary Figure 7: LC-MS analysis (selected *m/z* signals in the negative mode: 285.221, 301.217, 331.191, 329.175) of *in vitro* enzyme assays from microsomal preparations of indicated enzymes. The assays with the given enzymes were incubated with different substrates. Ferruginol (**4**), 11-hydroxyferruginol (**5**), CA (**9**) and CO (**10**). Note: Ferruginol is poorly detected by LC-MS, while 11-hydroxyferruginol is poorly detected by GC-MS.

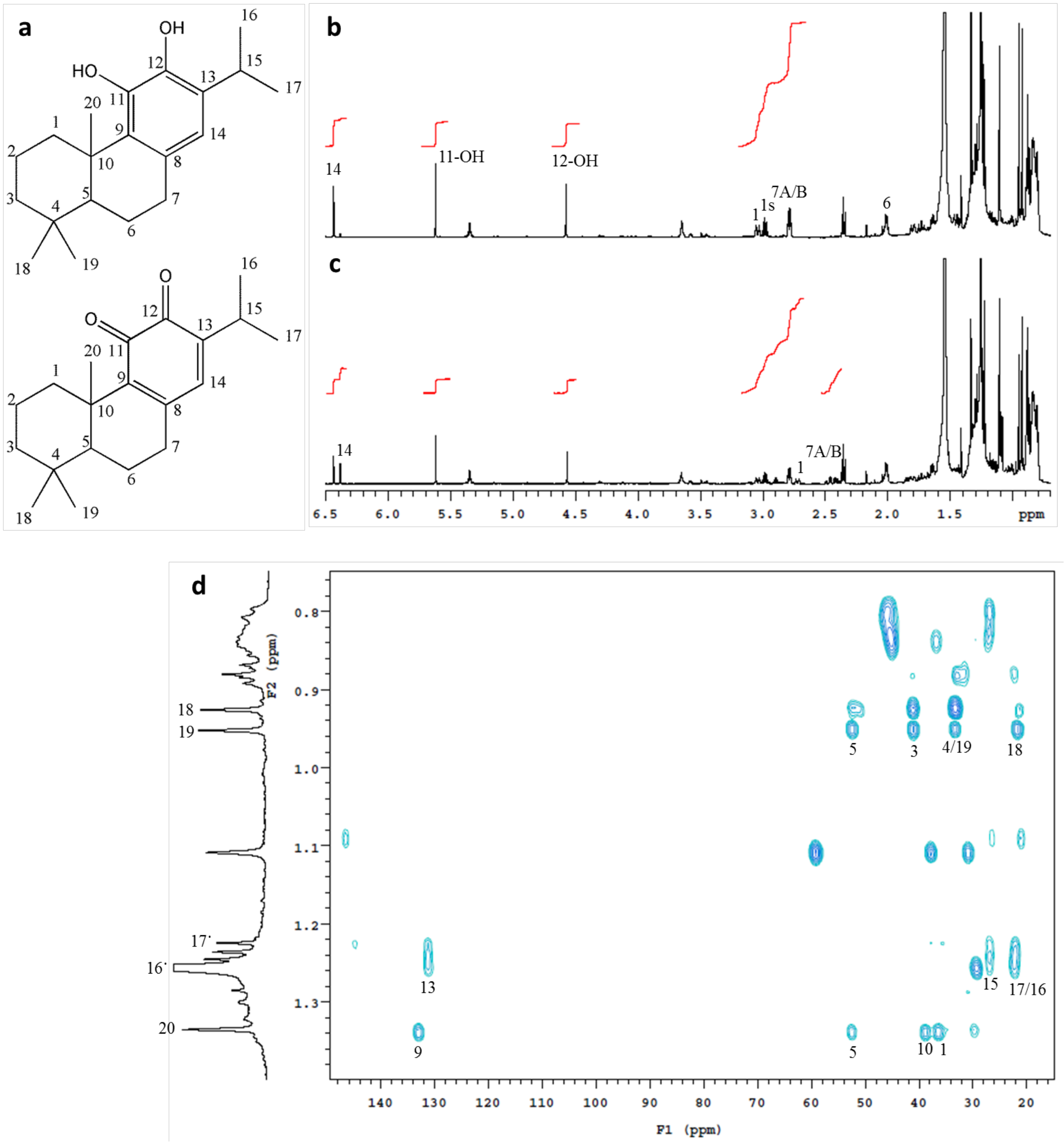
a
b
c
d
OH
HO
11-OH
12-OH
7A/B
1s
ppm
F2 (ppm)
F1 (ppm)
4/19
17/16

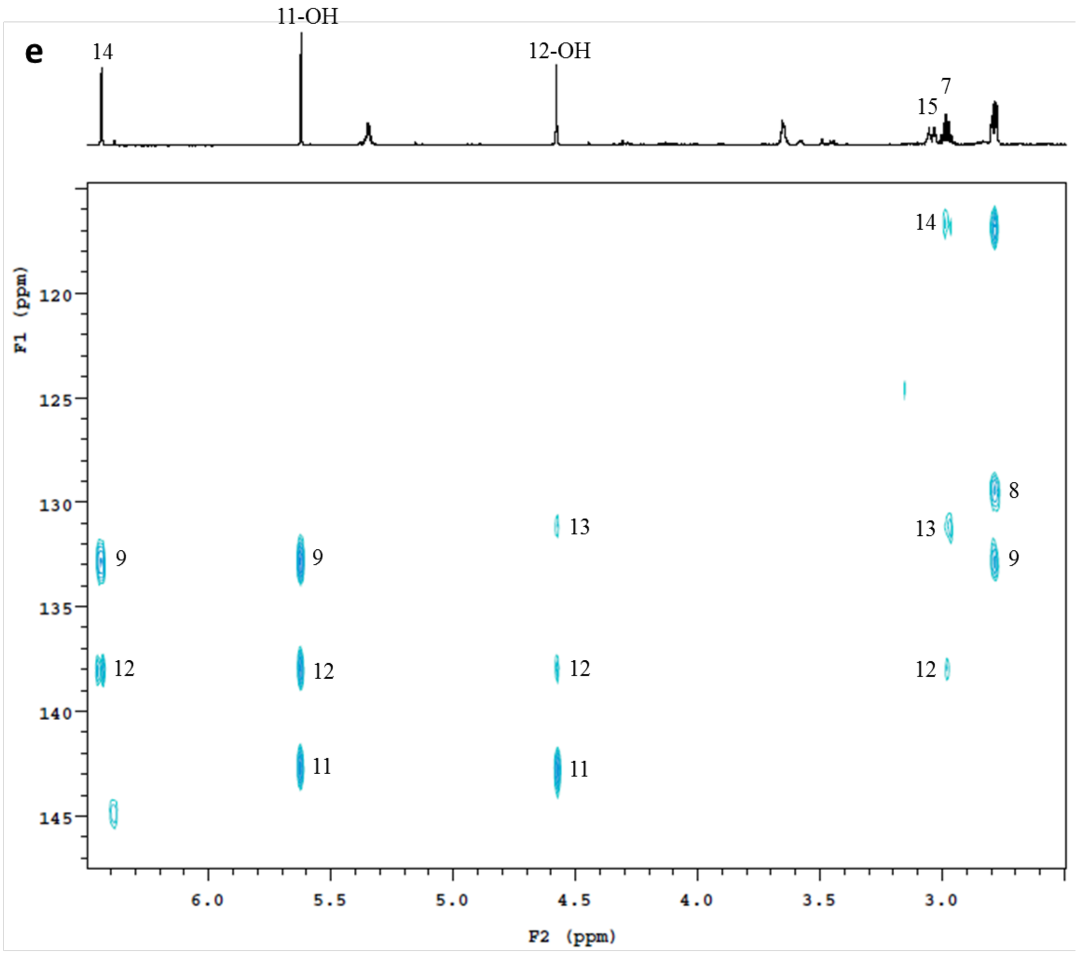
e
14
11-OH
12-OH
15
7
14
8
13
13
9
9
9
12
12
12
12
11
11
F1 (ppm)
F2 (ppm)

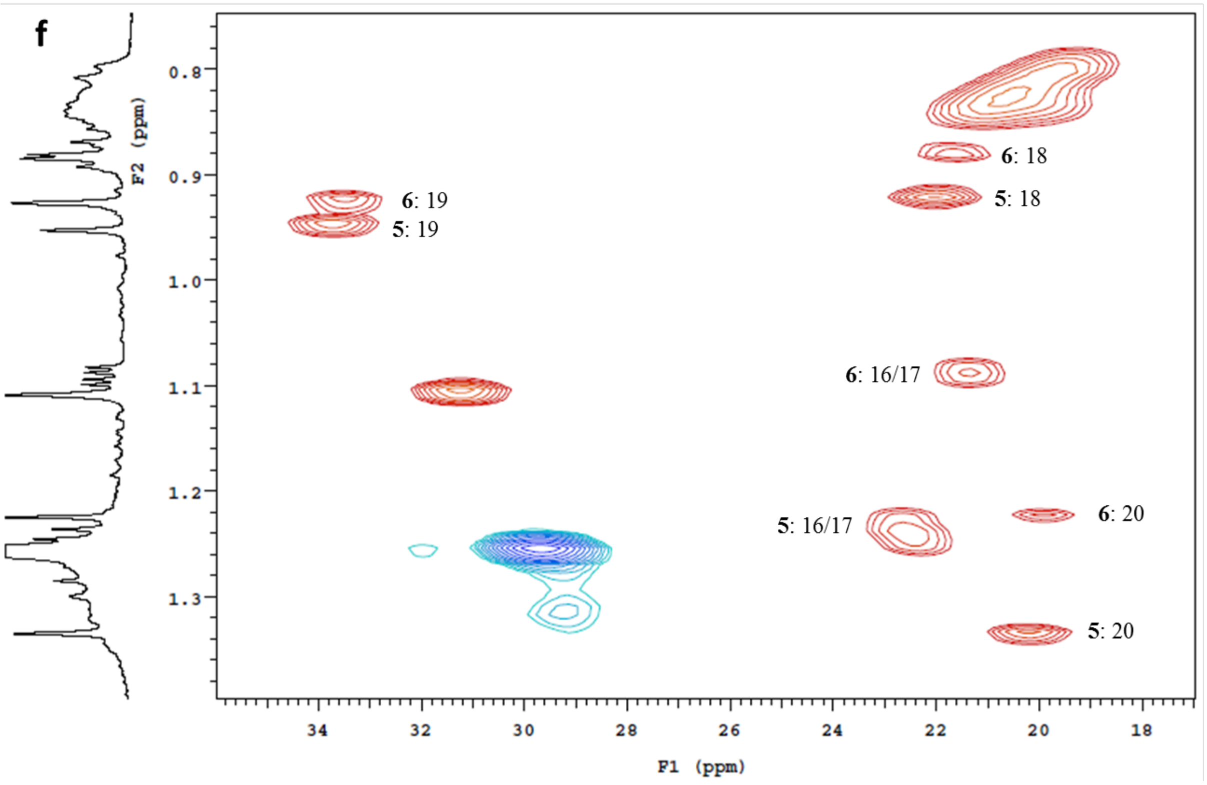
f
F2 (ppm)
6: 18
5: 18
6: 19
5: 19
6: 16/17
5: 16/17
6: 20
5: 20
F1 (ppm)

Supplementary Figure 8: NMR analysis of 11-hydroxyferruginol. (**a**) Structures of 11-hydroxyferruginol and hydroxyferruginol quinone with labeled carbon atoms. (**b**) ^{1}H NMR spectrum of 11-hydroxyferruginol immediately after dissolving in $CDCl_3$. (**c**) ^{1}H NMR spectrum of 11-hydroxyferruginol 24 h after dissolving in $CDCl_3$ showing signals of 11-hydroxyferruginol and hydroxyferruginol quinone. (**d**) Labeled: HMB correlations via $^{2}J_{CH}$ and $^{3}J_{CH}$ of methyl group proton signals of 11-hydroxyferruginol. Vertical trace: ^{1}H 1D NMR spectrum. (**e**) Low-field part of the HMBC spectrum of 11-hydroxyferruginol. Horizontal trace: ^{1}H 1D NMR spectrum. (**f**) Methyl group region of the HSQC spectrum of 11-hydroxyferruginol (5) after partial conversion to hydroxyferruginol quinone (6). Vertical trace: ^{1}H 1D NMR spectrum.

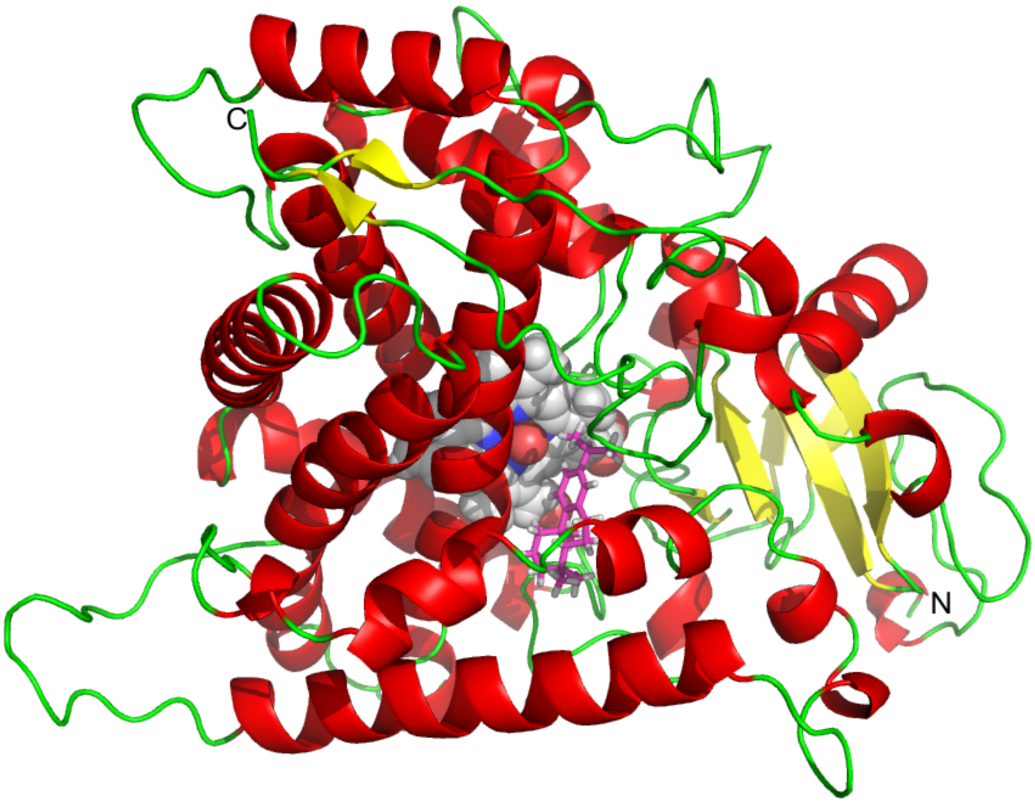

Supplementary Figure 9: 3D-model of CYP76AH1 as representative of all other FS and HFSs. The heme (space fill representation with orange colored iron ion) and the substrate (magenta carbon atoms) are located in the center.

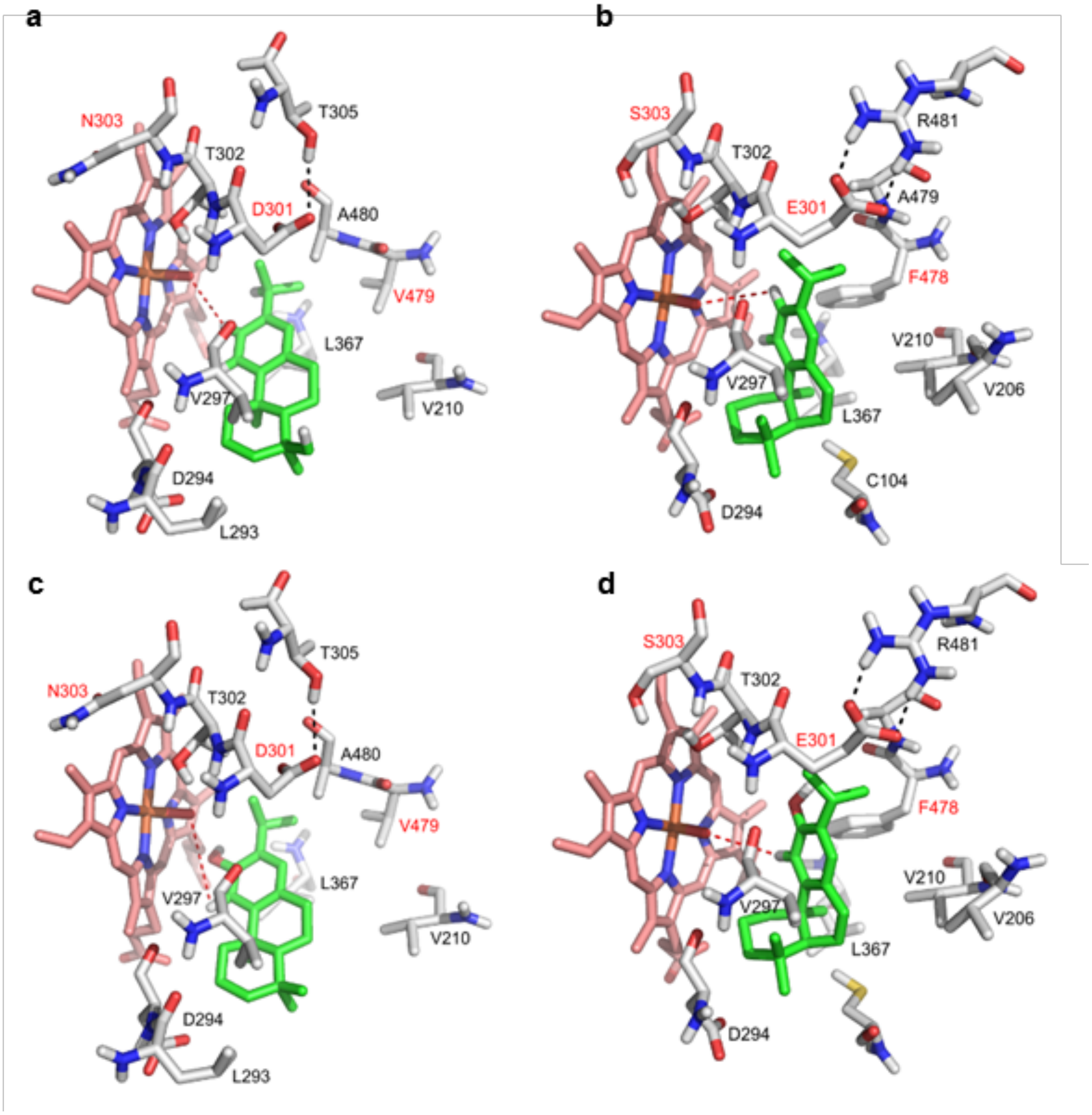

Supplementary Figure 10: 3D-model of the active sites of CYP76AH1 and CYP76AH22 with bound substrate (green carbon atoms) and heme (orange carbon atoms). Red labelled amino acid residues influence the product specificity which was proven by site directed mutagenesis. In the active center of the enzymes the substrate is mainly recognized by hydrophobic amino acid residues (V297, L367, V210 and V479 or F478, respectively). Active sites of (**a**) CYP76AH1 and (**b**) of CYP76AH22 with bound

abietatriene. The hydrogen atom of the aromatic ring system of the substrate is abstracted by the reactive oxygen atom bound to the iron ion. They are in closest proximity (3.6 Å in CYP76AH1 and 3.4 Å in CYP76AH22, red dashed line) which supports the oxidation reaction. (**c**) Active site of CYP76AH1 with bound ferruginol. The substrate cannot be efficiently oxidized because its distance to the reactive oxygen atom is 4.6 Å and therefore too far to support oxidation at C12 (red dashed line). In contrast, the substitution of V479 in CYP76AH1 by the more bulky F478 in CYP76AH22 leads to a slightly different docking arrangement of ferruginol (**d**). Therefore, the hydrogen atom at C12 can be abstracted by the reactive oxygen atom which is in a distance of 3.1 Å.

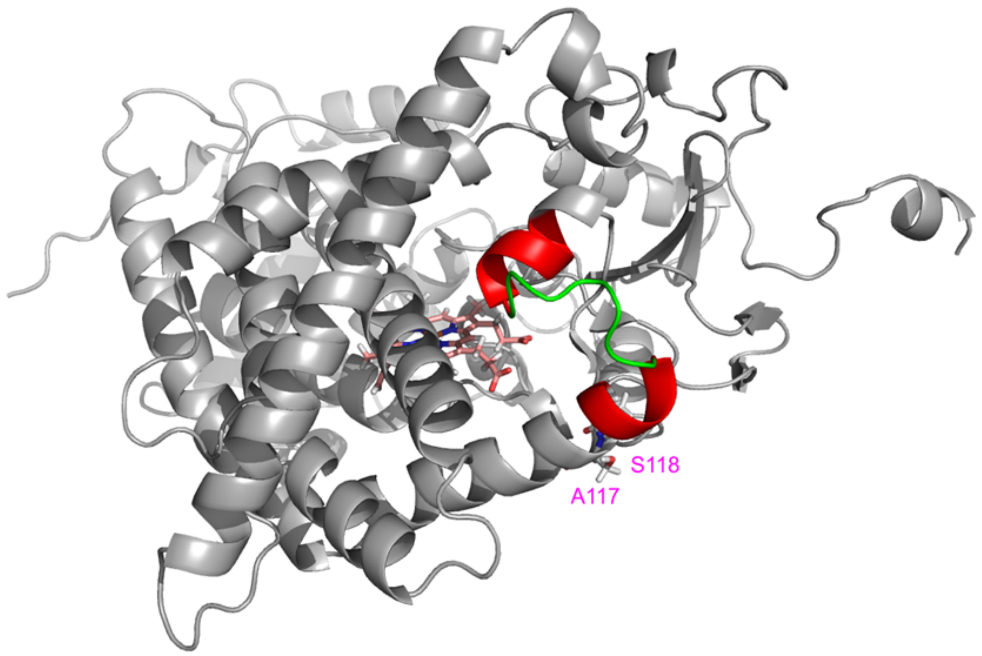

Supplementary Figure 11: 3D-model of CYP76AH1 with bound heme in the center. Labelled amino acids A117 and S118 are distinct from those in HFS. They may influence the secondary structure in the colored region and restrict access to the active site cleft.

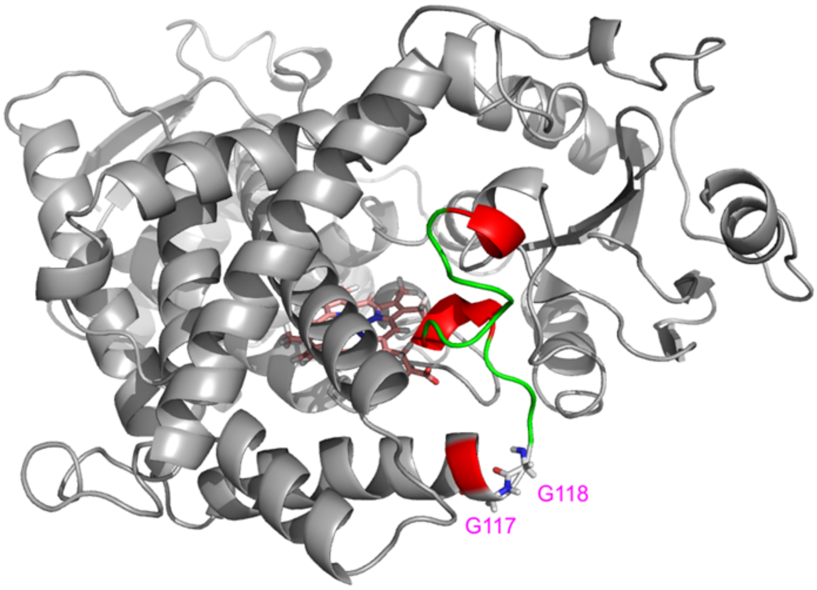

Supplementary Figure 12: 3D-model of CYP76AH22 with bound heme in the center.
Labelled amino acids G117 and G118 disturb the α-helix present in CYP76AH1 and influence the secondary structure in the highlighted region.

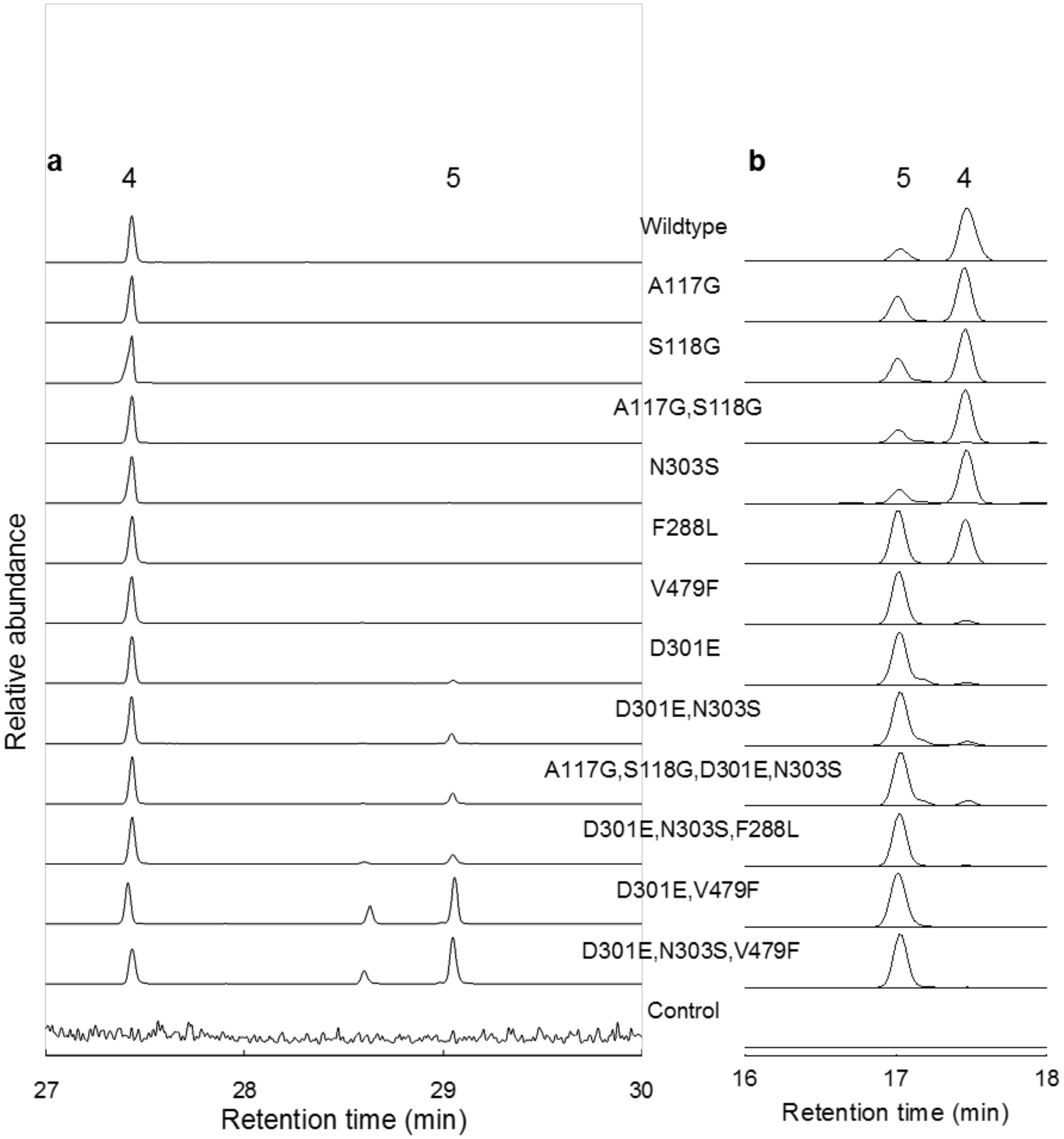

Supplementary Figure 13: Chromatographic analysis of extracts from yeast strains expressing wild type and mutagenized CYP76AH1. They were co-expressed in yeast with GGPPS, CPS, MiS and ATR1. Part of the chromatograms showing elution of ferruginol (**4**) and 11-hydroxyferruginol (**5**) from (**a**) GC-MS (selected *m/z* signals: 286, 300, 302) and (**b**) LC-MS analysis (selected *m/z* signals in the negative mode: 285.221, 301.217) are given.

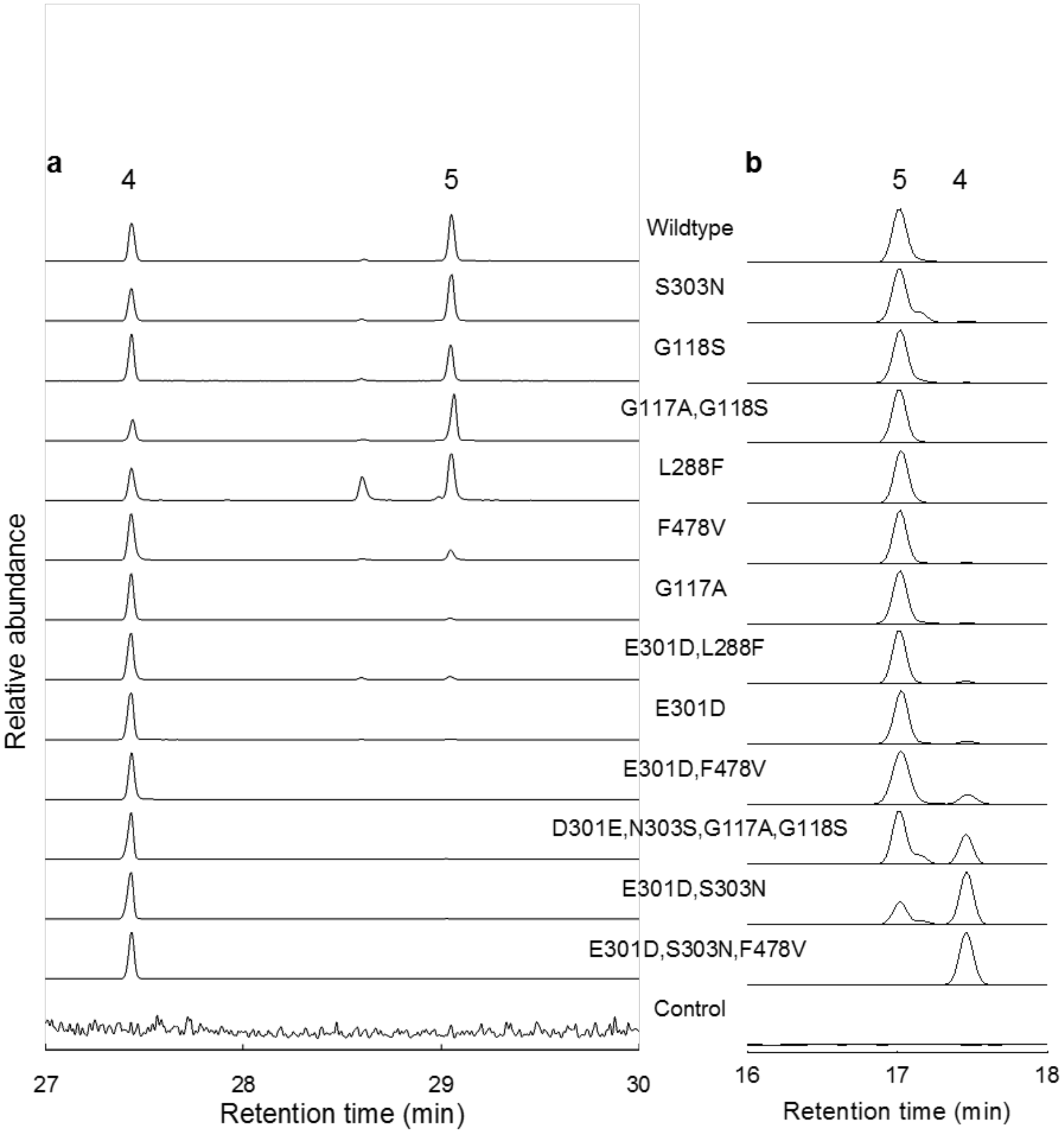

Supplementary Figure 14: Chromatographic analysis of extracts from yeast strains expressing wild type and mutagenized CYP76AH22. They were co-expressed in yeast with GGPPS, CPS, MiS and ATR1. Part of the chromatograms showing elution of ferruginol (**4**) and 11-hydroxyferruginol (**5**) from (**a**) GC-MS (selected *m/z* signals: 286, 300, 302) and (**b**) LC-MS analysis (selected *m/z* signals in the negative mode: 285.221, 301.217) are given.

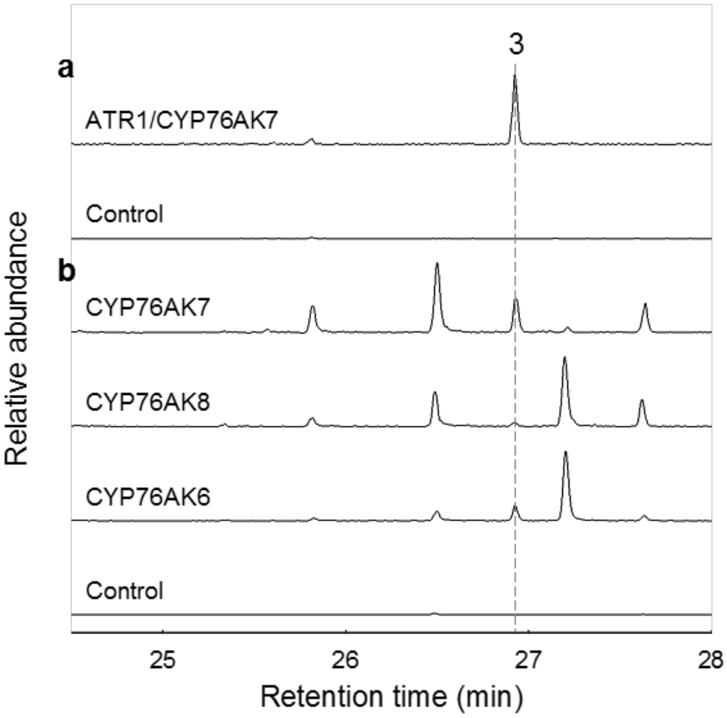

Supplementary Figure 15: GC-MS analysis of C_{20}Ox expression in yeast and *N. benthamiana* producing miltiradiene/abietatriene. Part of the GC-MS chromatogram containing miltiradien-20-al (3) is shown (selected *m/z* signal: 257). (**a**) Transient co-expression of CPS, MiS and indicated enzymes in *N. benthamiana*. (**b**) Co-expression of GGPPS, CPS, MiS, ATR1 and indicated CYPs in yeast.

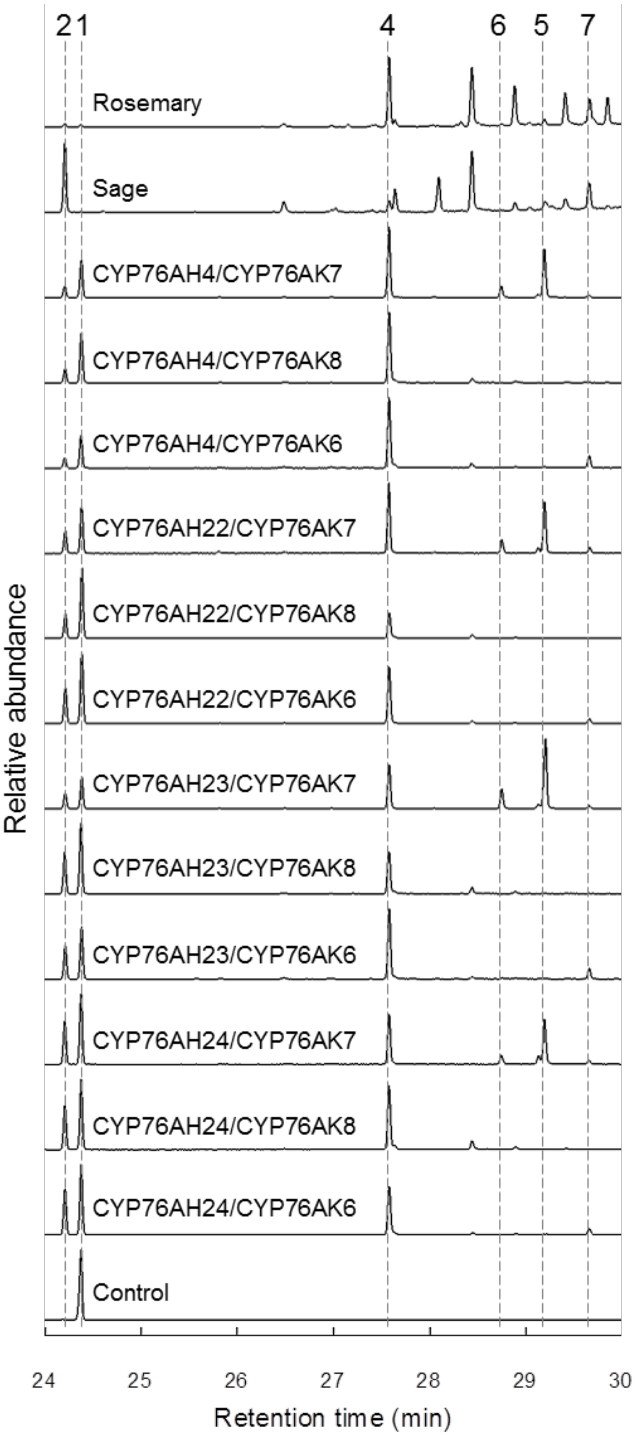

Supplementary Figure 16: GC-MS analysis of yeast strains co-expressing GGPPS, CPS, MiS, ATR1 and indicated CYPs. (Selected *m/z* signals: 270,272, 286, 300, 302, 316). Miltiradiene (**1**), abietatriene (**2**), ferruginol (**4**), 11-hydroxyferruginol (**5**), hydroxyferruginol quinone (**6**) and carnosaldehyde (**7**).

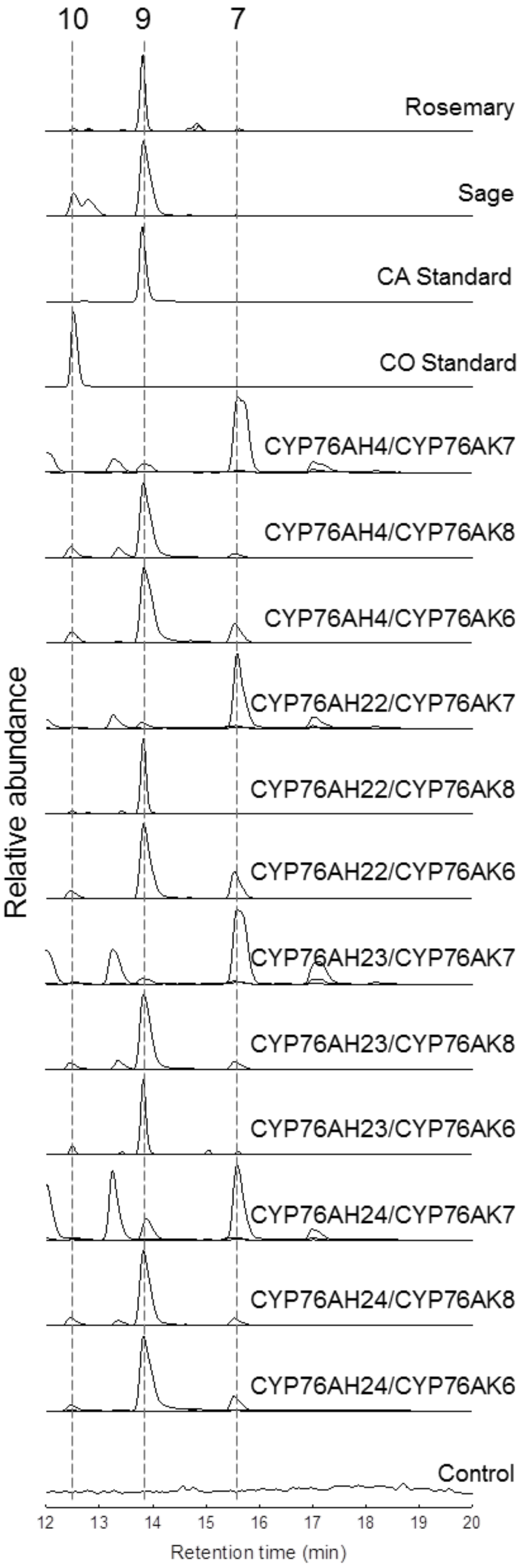
10
9
7
Rosemary
Sage
CA Standard
CO Standard
CYP76AH4/CYP76AK7
CYP76AH4/CYP76AK8
CYP76AH4/CYP76AK6
CYP76AH22/CYP76AK7
CYP76AH22/CYP76AK8
CYP76AH22/CYP76AK6
CYP76AH23/CYP76AK7
CYP76AH23/CYP76AK8
CYP76AH23/CYP76AK6
CYP76AH24/CYP76AK7
CYP76AH24/CYP76AK8
CYP76AH24/CYP76AK6
Control
Relative abundance
12 13 14 15 16 17 18 19 20
Retention time (min)

Supplementary Figure 17: LC-MS analysis of extracts obtained from rosemary and sage leaves, authentic standards and yeast strains expressing GGPPS, CPS, MiS, ATR1 and indicated CYPs. (Selected *m/z* signals in the negative mode: 315.196, 331.191, 329.175). Carnosaldehyde (7), CA (9) and CO (10).

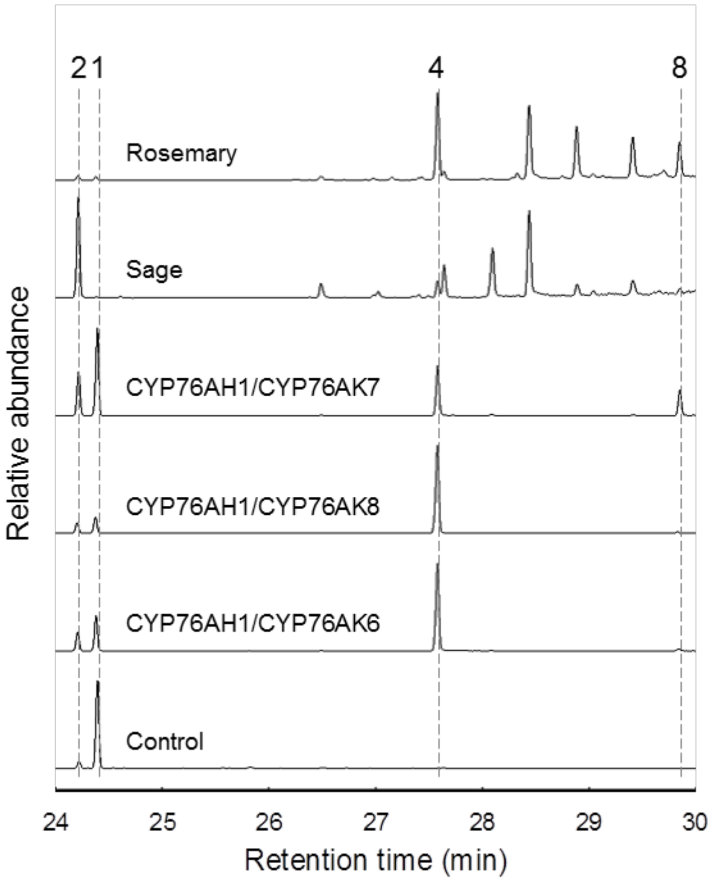

Supplementary Figure 18: Part of the GC-MS chromatogram of extracts of rosemary and sage as well as of yeast strains co-expressing GGPPS, CPS, MiS, ATR1 and indicated CYPs. (Selected *m/z* signals: 270, 272, 286, 300). Miltiradiene (**1**), abietatriene (**2**), ferruginol (**4**) and pisiferal (**8**).

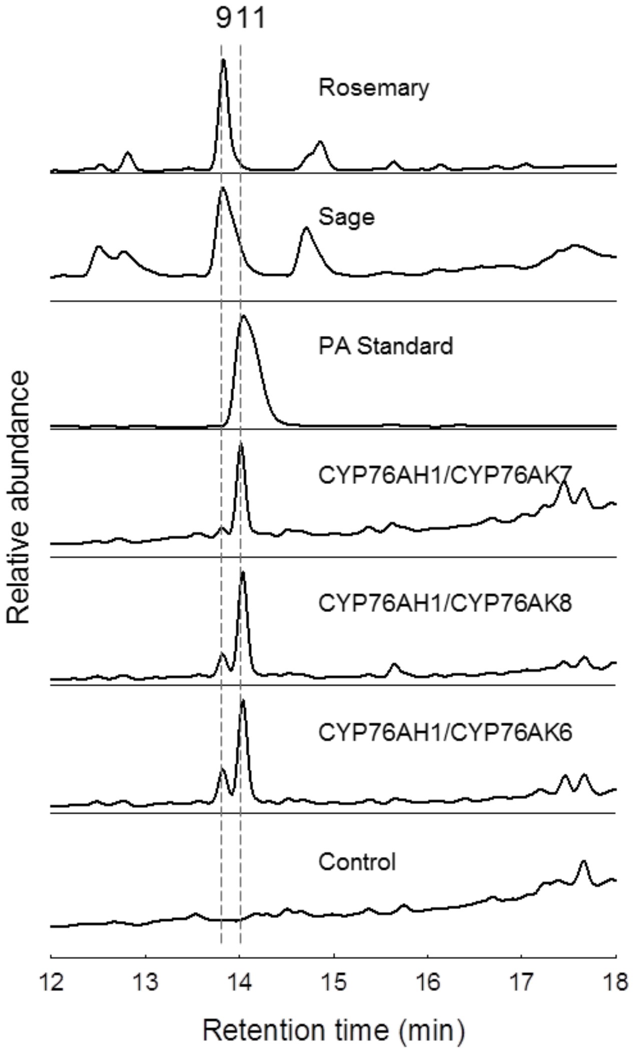

Supplementary Figure 19: LC-MS total ion chromatogram of extracts from rosemary, sage and from yeast strains co-expressing GGPPS, CPS, MiS, ATR1 and indicated CYPs. CA (9) and PA (11).

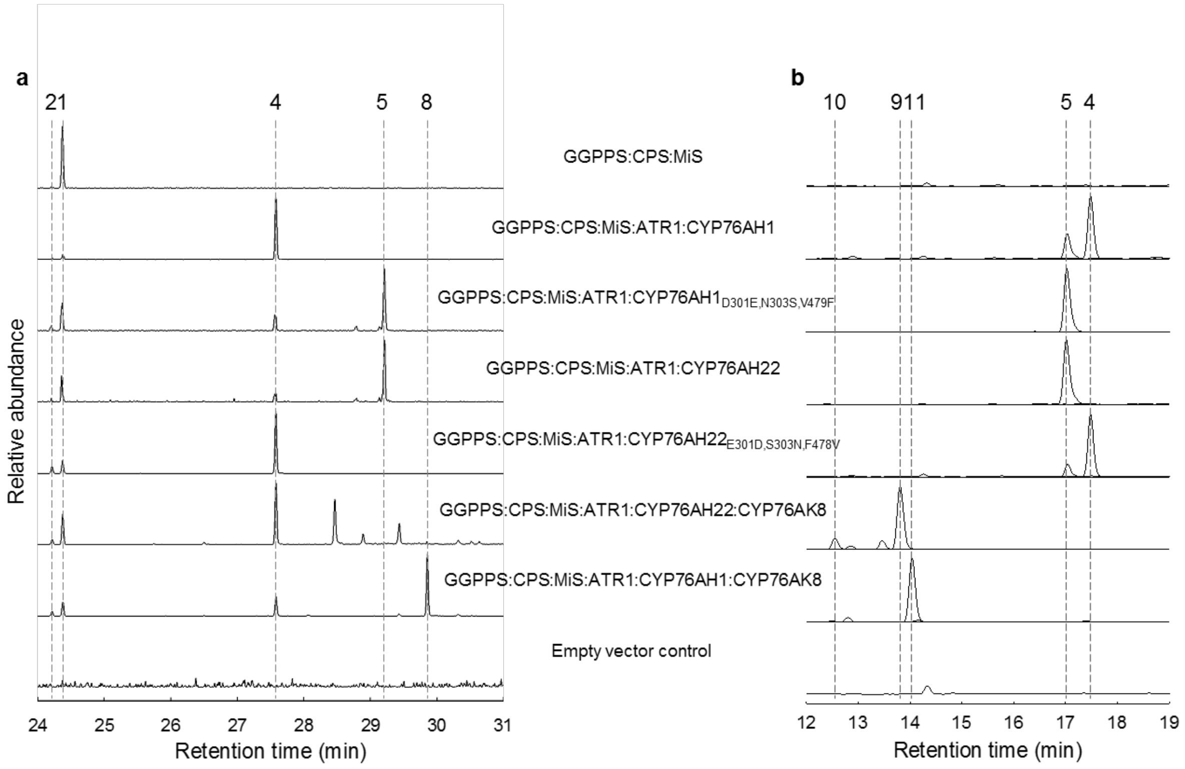

Supplementary Figure 20: Chromatographic analysis of engineered yeast strains which were used for absolute NMR quantification. (**a**) GC-MS analysis of indicated strains (selected *m/z* signals: 270, 272, 286, 300, 302, 316). (**b**) LC-MS analysis of yeast strains expressing the indicated enzymes (selected *m/z* signals in the negative mode: 285.221, 301.217, 315.196, 331.191, 329.175). Miltiradiene (**1**), abietatriene (**2**), ferruginol (**4**), 11-hydroxyferruginol (**5**), pisiferal (**8**), CA (**9**), CO (**10**) and PA (**11**).

a

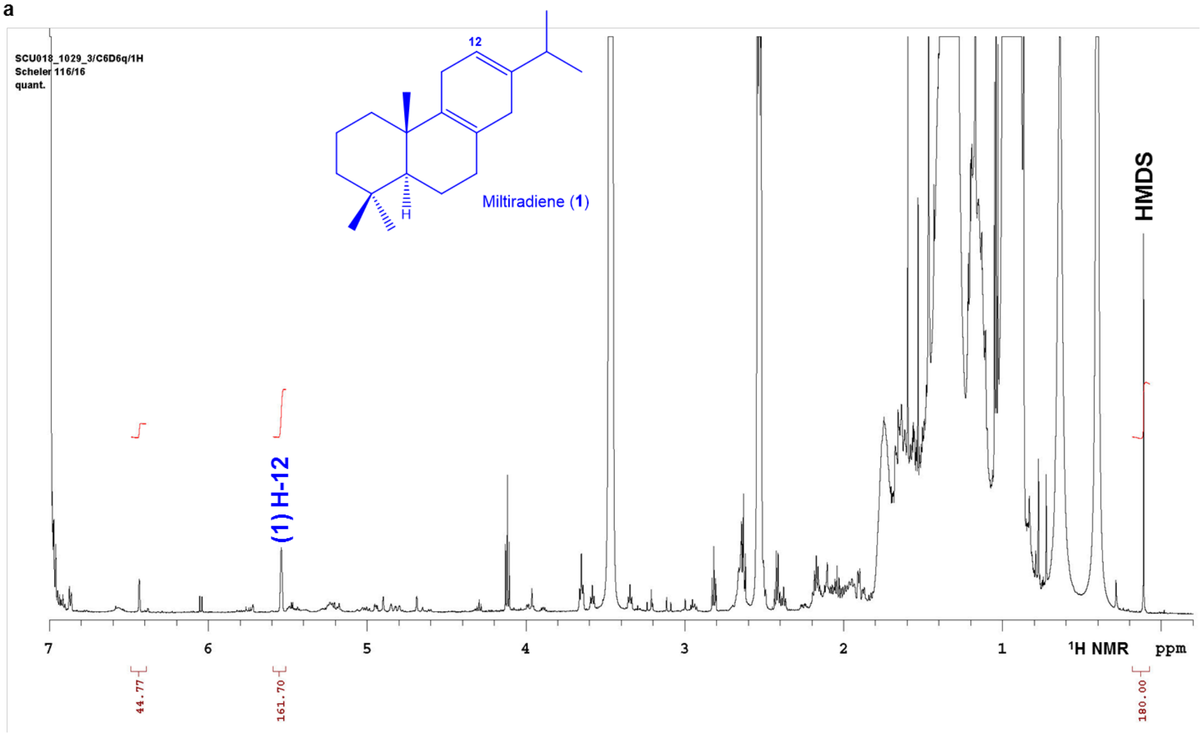

SCU018_1029_3/C6D6q/1H
Scheler 116/16
quant.
12
Miltiradiene (1)
(1) H-12
HMDS
7
6
5
4
3
2
1
^{1}H NMR
ppm
44.77
161.70
180.00

b

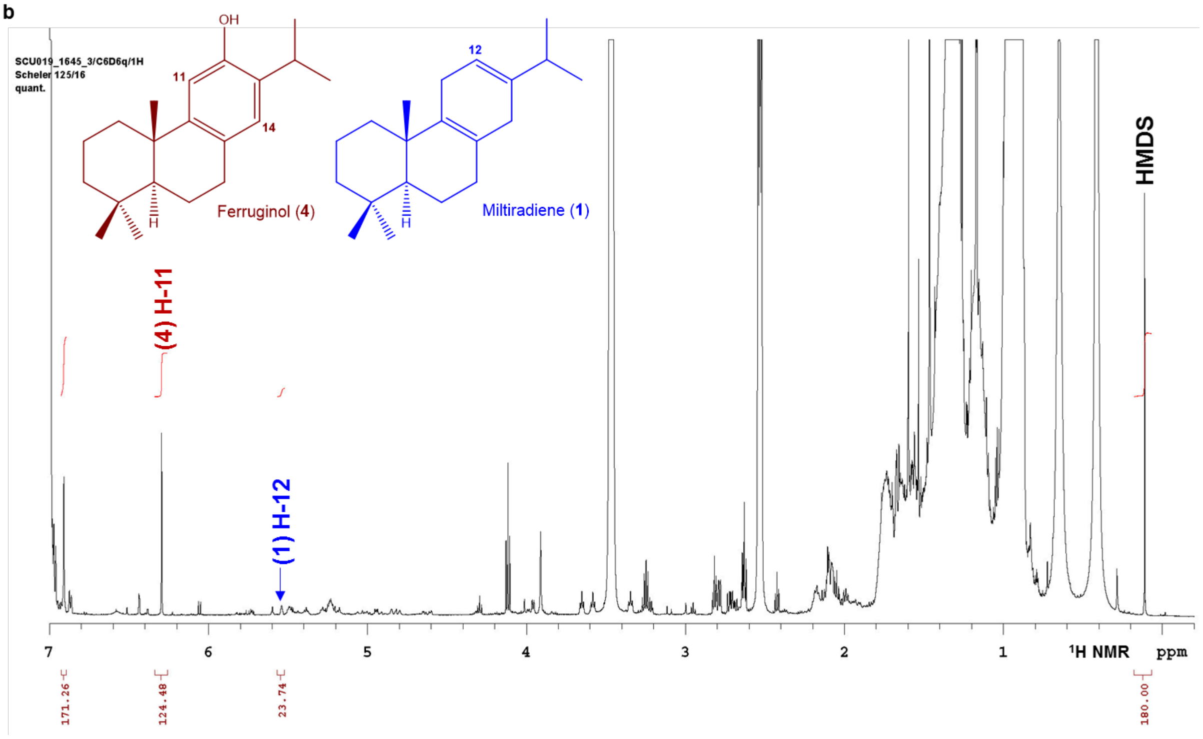

SCU019_1645_3/C6D6q/1H
Scheler 125/16
quant.
OH
11
14
Ferruginol (4)
12
Miltiradiene (1)
(4) H-11
(1) H-12
HMDS
7
6
5
4
3
2
1
^{1}H NMR
ppm
171.26
124.48
23.74
180.00

c

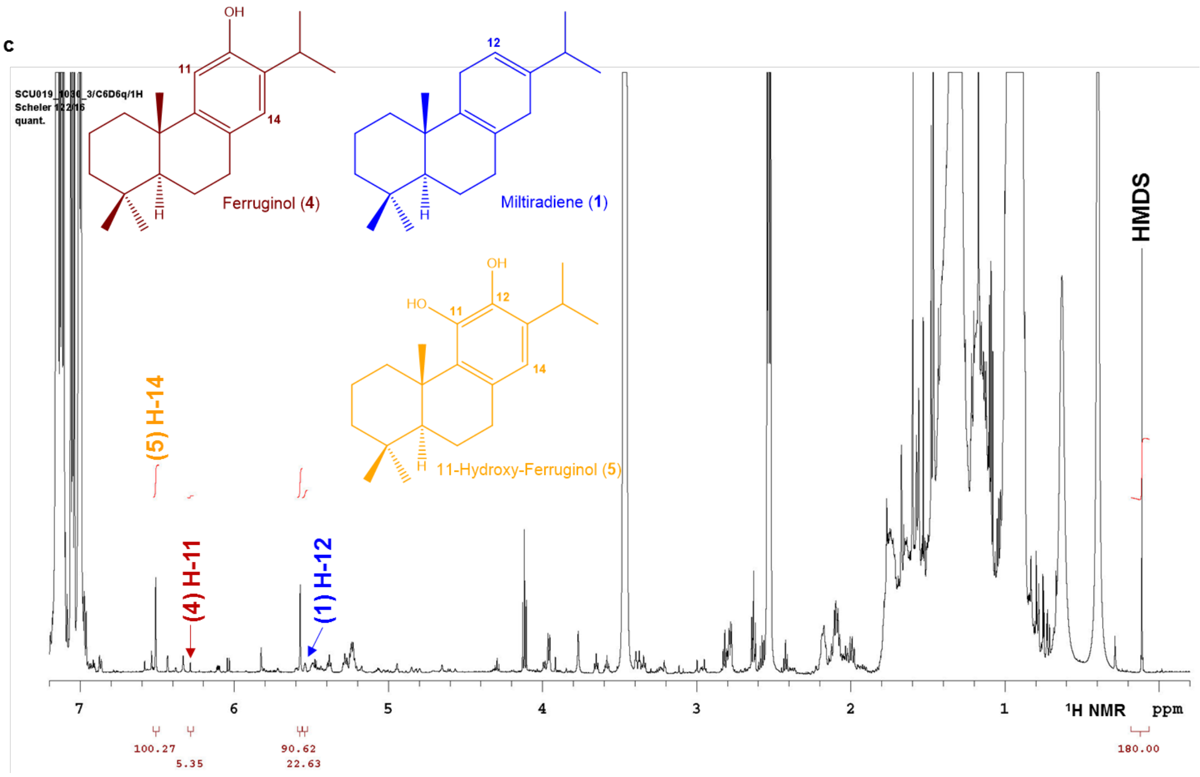

SCU019_1036_3/C6D6q/1H
Scheler 122/16
quant.
OH
11
14
Ferruginol (4)
12
Miltiradiene (1)
HO
11
12
OH
14
11-Hydroxy-Ferruginol (5)
(5) H-14
(4) H-11
(1) H-12
HMDS
7
6
5
4
3
2
1
1H NMR
ppm
100.27
5.35
90.62
22.63
180.00

d

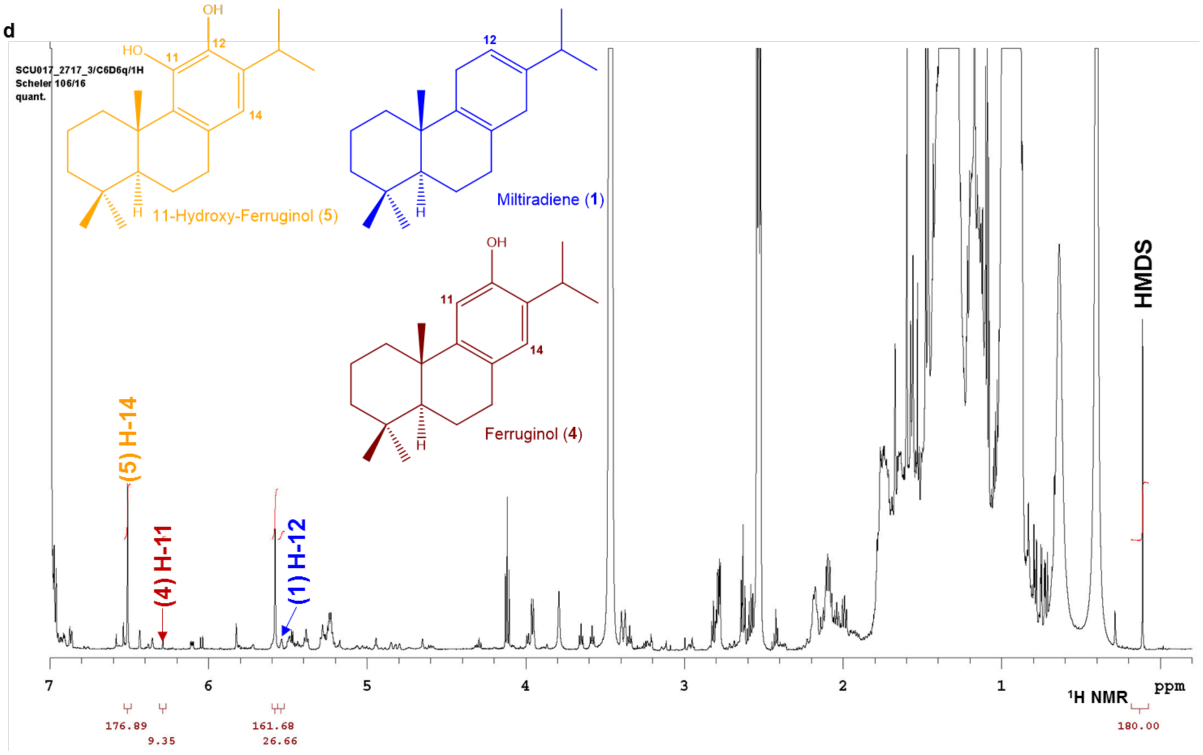

SCU017_2717_3/C6D6q/1H
Scheler 106/16
quant.
HO
11
OH
12
14
11-Hydroxy-Ferruginol (5)
12
Miltiradiene (1)
OH
11
14
Ferruginol (4)
(5) H-14
(4) H-11
(1) H-12
HMDS
7
6
5
4
3
2
1
1H NMR
ppm
176.89
9.35
161.68
26.66
180.00

e

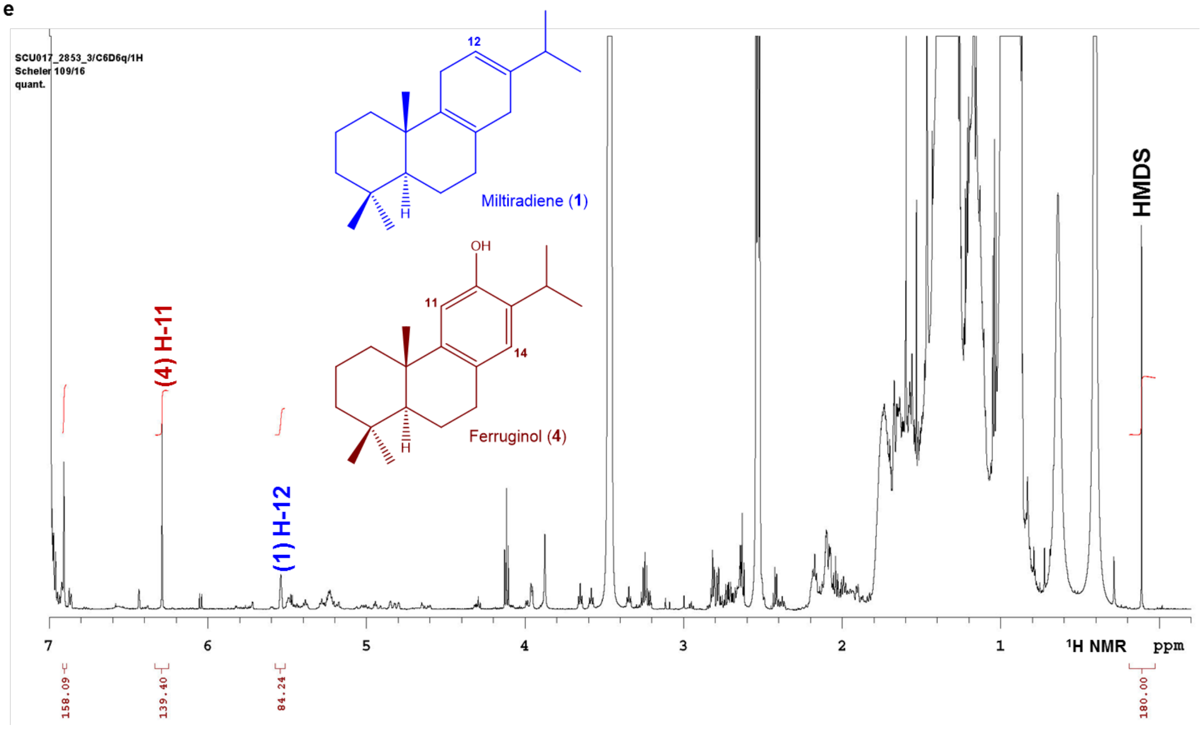

SCU017_2853_3/C6D6q/1H
Scheler 109/16
quant.
12
Miltiradiene (1)
OH
11
14
Ferruginol (4)
(4) H-11
(1) H-12
HMDS
7
6
5
4
3
2
1
1H NMR
ppm
158.09
139.40
84.24
180.00

f

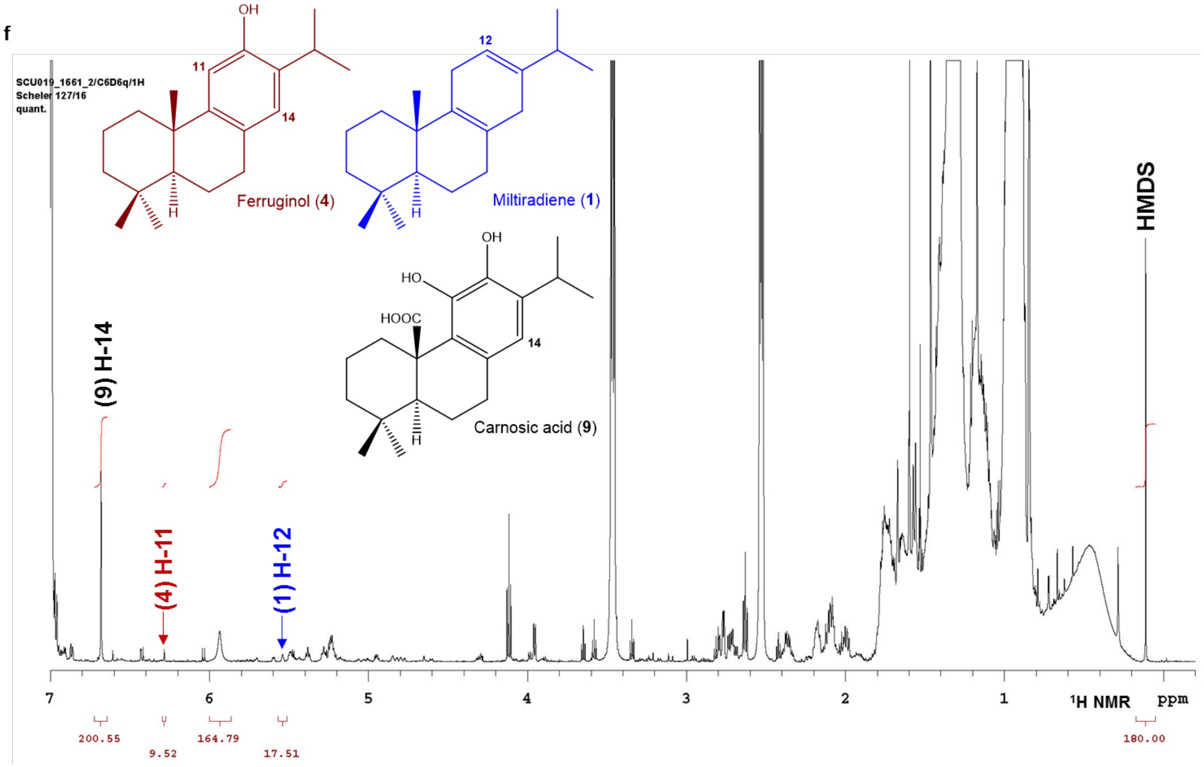

SCU019_1661_2/C6D6q/1H
Scheler 127/16
quant.
OH
11
14
Ferruginol (4)
12
Miltiradiene (1)
OH
HO
HOOC
14
Carnosic acid (9)
(9) H-14
(4) H-11
(1) H-12
HMDS
7
6
5
4
3
2
1
1H NMR
ppm
200.55
9.52
164.79
17.51
180.00

g

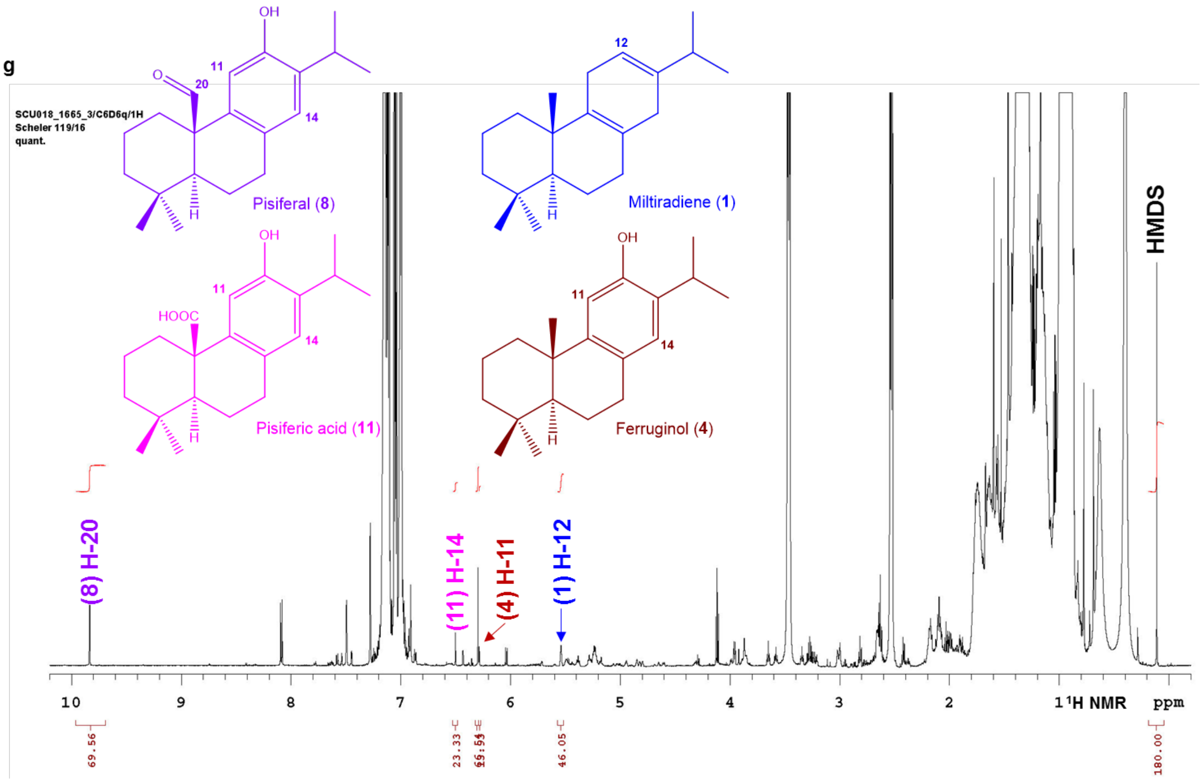

Supplementary Figure 21: ^{1}H NMR spectra of engineered yeast for quantification. The strains expressed the enzymes GGPPS, CPS, MiS (**a**) together with ATR1 and CYP76AH1 (**b**), CYP76AH22 (**c**), CYP76AH1$_{D301EN303SV479F}$ (**d**), CYP76AH22$_{E301DS303NF478V}$ (**e**), CYP76AH22 and CYP76AK8 (**f**), or CYP76AH1 and CYP76AK8 (**g**). Product specific signals, which were used for quantification, are highlighted. The corresponding integrals including the peak areas are given. As internal standard hexamethyldisiloxane (HMDS) was used for quantification.

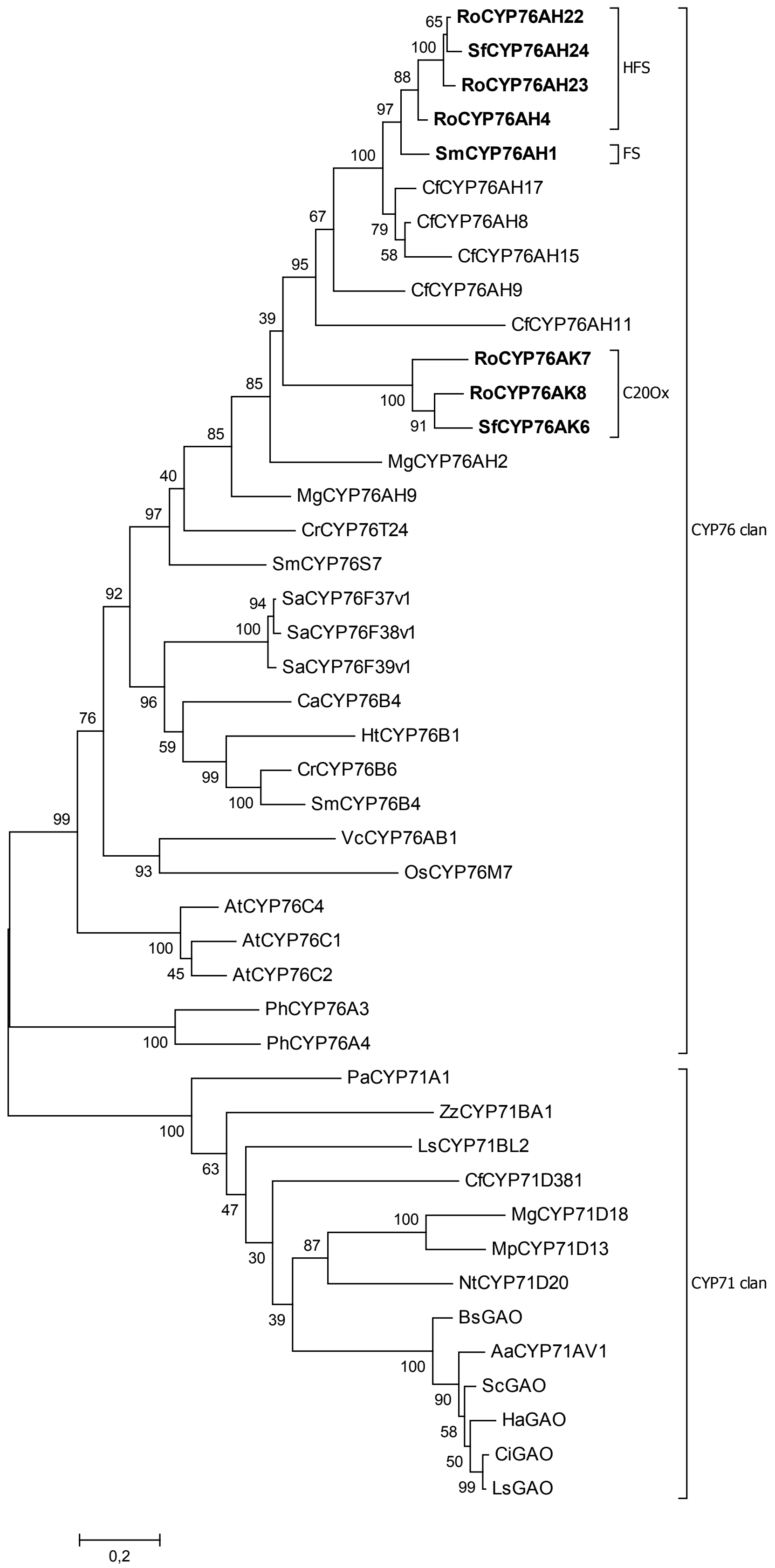
RoCYP76AH22
SfCYP76AH24
RoCYP76AH23
RoCYP76AH4
SmCYP76AH1
CfCYP76AH17
CfCYP76AH8
CfCYP76AH15
CfCYP76AH9
CfCYP76AH11
RoCYP76AK7
RoCYP76AK8
SfCYP76AK6
MgCYP76AH2
MgCYP76AH9
CrCYP76T24
SmCYP76S7
SaCYP76F37v1
SaCYP76F38v1
SaCYP76F39v1
CaCYP76B4
HtCYP76B1
CrCYP76B6
SmCYP76B4
VcCYP76AB1
OsCYP76M7
AtCYP76C4
AtCYP76C1
AtCYP76C2
PhCYP76A3
PhCYP76A4
PaCYP71A1
ZzCYP71BA1
LsCYP71BL2
CfCYP71D381
MgCYP71D18
MpCYP71D13
NtCYP71D20
BsGAO
AaCYP71AV1
ScGAO
HaGAO
CiGAO
LsGAO
HFS
FS
C20Ox
CYP76 clan
CYP71 clan
0,2

Supplementary Figure 22: Phylogenetic analysis of CYPs involved in the biosynthesis of CA and PA. The maximum likelihood tree was generated using MEGA (version 6) from protein sequence alignment and illustrates the relationship of CYP enzymes of the PD biosynthesis to other proteins from the CYP76 and CYP71 clan. Bootstrap values (1000 repeats) are indicated for each branch of the tree.

Supplementary Tables

Supplementary Table 1: Primers used for cloning, introduction of single point mutations and quantitative real-time PCR.

Gene	Application	Primer ID	Sequence
CYP76AH22	Cloning	RoFS1_for1	TTTGAAGACAAAATGGATTCTTTTCCTCTT
		RoFS1_rev1	TTTGAAGACAGACTTGCCCGTATTTGTG
		RoFS1_for2	TTTGAAGACGCAAGTCTTTTCCGGCAG
		RoFS1_rev2	TTTGAAGACTCCATGCTCTGATGCGAGA
		RoFS1_for3	TTTGAAGACAGCATGGAAGATAGCCAGT
		RoFS1_rev3	TTTGAAGACTTCAACTTGTTGTCGTTCGT
		RoFS1_for4	TTTGAAGACAAGTTGAAGATCGATCACCT
	Introduction of single point mutation	RoFS1E301D_for	GTTTGTTGGAGGATCTGACACAAGCACGACAGAGATCG
		RoFS1E301D_rev	CGATCTCTGTCGTGCTTGTGTCAGATCCTCCAACAAAC

		RoFS1E301DS303N_for	GTTTGTTGGAGGATCTGACACAAACACGACAGAGATCGAG
		RoFS1E301DS303N_rev	CTCGATCTCTGTCGTGTTTGTGTCAGATCCTCCAACAAAC
		RoFS1G117A_for	GGTTCCTCCCCGTGGCGGGCGAGTGGCGCG
		RoFS1G117A_rev	CGCGCCACTCGCCCGCCACGGGGAGGAACC
		RoFS1G117AG118S_for	GGTTCCTCCCCGTGGCGAGCGAGTGGCGCG
		RoFS1G117AG118S_rev	CGCGCCACTCGCTCGCCACGGGGAGGAACC
		RoFS1G118S_for	CCTCCCCGTGGGGAGCGAGTGGCGCG
		RoFS1G118S_rev	CGCGCCACTCGCTCCCCACGGGGAGG
		RoFS1S303N_for	GGAGGATCTGAAACAAACACGACAGAGATCGAGTGG
		RoFS1S303N_rev	CCACTCGATCTCTGTCGTGTTTGTTTCAGATCCTCC
		AH22F478V_for	GGTGTGTTGTTTGGCGTTGCGGTGCGGAGGG

		AH22F478V_rev	CCCTCCGCACCGCAACGCCAAACAACACACC
		AH22L288F_for	GTTGAAGATCGATCACTTCACACATCTCATGCTGG
		AH22L288F_rev	CCAGCATGAGATGTGTGAAGTGATCGATCTTCAAC
CYP76AH23	Cloning	RoFS1_for1	TTTGAAGACAAAATGGATTCTTTTCCTCTT
		RoFS2_rev1	TTTGAAGACTTGCCCGTATTTGTGCATGA
		RoFS2_for2	TTTGAAGACACGGGCAAGTATTCTCCG
		RoFS2_rev2	TTTGAAGACTCCATGCTCTGATGCGAG
		RoFS2_for3	TTTGAAGACAGCATGGAAGATAGTCAGG
		RoFS2_rev3	TTTGAAGACTCAACTTACAGTCGTTGG
		RoFS2_for4	TTTGAAGACTAAGTTGAAGATGGATCACC
		RoFS2_rev4	TTTGAAGACAAAAGCTTATGCCTTAAAGGG

CYP76AH4	Cloning	CYP76AH4_for1neu	TTTGAAGACAAAATGGATTCCTTTCCTCTTCTCGTTGCTCTCTTCTTCATCGCTGTGACAACTTTCCTT
		CYP76AH4_for2	TTTGAAGACTACGGGCAAGTATTCTCCGG
		CYP76AH4_for3	TTTGAAGACGCATGGAAGATAGCCAGTGG
		CYP76AH4_for4	TTTGAAGACAAGTTGAAAACCGATCACCT
		CYP76AH4_rev1	TTTGAAGACTGCCCGTATTTGTGCATGATT
		CYP76AH4_rev2	TTTGAAGACTTCCATGCTCTGATGCGAGA
		CYP76AH4_rev3	TTTGAAGACTTCAACTTGTTGTCGTTCGT
		CYP76AH4_rev4_2	TTTGAAGACAAAAGCTCAAGACTTAACTATTGGGA
CYP76AH1	Introduction of single point mutation	CYP76AH1D301E_f	CGTGGGAGGATCGGAAACGAACACGACCTCG
		CYP76AH1D301E_r	CGAGGTCGTGTTCGTTTCCGATCCTCCCACG
		CYP76AH1A117G_for	GGTTCCTCCCCGTGGGCAGCGAGTGGCGCG

		CYP76AH1A117G_rev	CGCGCCACTCGCTGCCCACGGGGAGGAACC
		CYP76AH1A117GS118G_for	GGTTCCTCCCCGTGGGCGGCGAGTGGCGCGACATGC
		CYP76AH1A117GS118G_rev	GCATGTCGCGCCACTCGCCGCCCACGGGGAGGAACC
		CYP76AH1D301EN303S_for	CGTGGGAGGATCGGAAACGAGCACGACCTCGATCG
		CYP76AH1D301EN303S_rev	CGATCGAGGTCGTGCTCGTTTCCGATCCTCCCACG
		CYP76AH1N303S_for	GGAGGATCGGACACGAGCACGACCTCGATCG
		CYP76AH1N303S_rev	CGATCGAGGTCGTGCTCGTGTCCGATCCTCC
		CYP76AH1S118G_for	GGTTCCTCCCCGTGGCCGGCGAGTGGCGCG
		CYP76AH1S118G_rev	CGCGCCACTCGCCGGCCACGGGGAGGAACC
		AH1F288L_for	GTTGAAAACGCATCACTTAACCCATCTCATGCTGG
		AH1F288L_rev	CCAGCATGAGATGGGTTAAGTGATGCGTTTTCAAC

		AH1V479F_for	GCGAGTTGTTTGGGTTTGCCGTGCGCAGGGC
		AH1V479F_rev	GCCCTGCGCACGGCAAACCCAAACAACTCGC
CYP76AK7	Amplification from cDNA	Ro34450-fw	CACCATGGATGCTTTTGTTGTTTTCTCCCTG
		Ro34450-rv	TCAAACCTTGATGGGTTTAGCCCTAAGTG
	Golden Gate Cloning	CYP34450 For1	TTTGAAGACAAAATGGATGCTTTTGTTGTTTTCTC
		CYP34450 Rev1	TTTGAAGACCGGAGTTGTAAGATGTTGCC
		CYP34450 For2	TTTGAAGACCAACTCCGCGGTGACCCCCAC
		CYP34450 Rev2	TTTGAAGACGAGGTCGGTCATGGTGGTGAA
		CYP34450 For3	TTTGAAGACCCGACCTCGTTTTCTCGAC
		CYP34450 Rev3	TTTGAAGACAAAAGCTCAAACCTTGATGGGTTTAGC
	qPCR analysis	Roff34450-For1256	ACGCAGATAAATGGCTATACAATCC
		Roff34450-Rev1535	ATTCAGCCCCTCCTTCAAGTTCC
CYP76AK8	Amplification from cDNA	Ro33941-fw	CACCATGCAATTGTTCATTATTCTATCCCTAG
		Ro33941-rv	TCAAACCTTGATGGGAACAGCCCTTAG

	Golden Gate Cloning	CYP33941_For1	TTTGAAGACAAAATGCAATTGTTCATTATTCTATCCCTAG
		CYP33941_Rev1	TTTGAAGACTCTCGGCCAAGTTGTAAGATG
		CYP33941_For2	TTTGAAGACGGCCGAGATCCCCACAAG
		CYP33941_Rev2	TTTGAAGACGGCGACGAAACCACCACG
		CYP33941_For3	TTTGAAGACTCGTCGCCGGAAATGGCGAG
		CYP33941_Rev3	TTTGAAGACAAAAGCTCAAACCTTGATGGGAACAGC
	qPCR analysis	Roff33941-For1791	TGGATAGCGAGATTGATTTTGGAG
		Roff33941-Rev1967	TCTGGAGTTGTTTGGATTCTGCC
CYP76AK6	Amplification from cDNA	0850-fw	CACCATGCAAGTTCTCATCCTTCTTTCTCT
		0850-rev	TCAAACTTTGATGGGAATAGCTCTTAG
	Golden Gate Cloning	SfruCYP850 For1	TTTGAAGACAAAATGCAAGTTCTCATCCTTCTTTCTCTGGCC
		SfruCYP850 Rev1	TTTGAAGACGCACTGCGATCGGAGCGCACGGCGTCGAAAAG
		SfruCYP850 For2	TTTGAAGACCGCAGTGCAAATACTTGGCCACGGCGAGGTTTCTA
		SfruCYP850 Rev2	TTTGAAGACAAAAGCTCAAACTTTGATGGGAATAGCTCTTAGG
	qPCR analysis	For811-Sfru850/2	AGCTCGAAACACGGCGACTTACC

		Rev1067-Sfru850/2	ATTCTTCGATGATGCTTTTGTCTCC
Salvia fruticosa eIF-4a	qPCR - reference gene	For-eIF-4A-Sfru	TTGTTGCCATTGACATCTTCACTT
		Rev-eIF-4A-Sfru	CTCTCCCATGGCTGACATAACACT
Rosmarinus officinalis eIF-4a	qPCR - reference gene	Roff21732/35elf4a-For221	ACGAGATGGGAATAAAGGAGGAG
		Roff21732/35elf4a-Rev546	ATCCTCACCCACACTTTTGCCTC
pGal_syn1	Primer extension PCR	pGal_syn1_1_for	TTTGAAGACAAGGAGATTACTATCCCGGATTAGAA
		pGal_syn1_1_rev	TTTGAAGACACCATTTGTTTTTTTTTATGACGTTAAGCT
pGal_syn3	Primer extension PCR	pGal_syn1_3_for	TTTGAAGACAAGGAGAGAGTGTGGTCGGATTAGAA
		pGal_syn1_3_rev	TTTGAAGACACCATTTGTTTTTTTTTATGATGTTAATGT
pGal_syn4	Primer extension PCR	pGal_syn1_4_for	TTTGAAGACAAGGAGTTGTGTTGTTCGGATTAGAAG
		pGal_syn1_4_rev	TTTGAAGACACCATTTGTTTTTTTCTTTGACGTTAAGCT
pGal_syn5	Primer extension PCR	pGal_syn1_5_for	TTTGAAGACAAGGAGGGCTTAGCGTCGGATTAGAAG
		pGal_syn1_5_rev	TTTGAAGACACCATTTTTTTTTTTTTATGATGTTAAGGT
pGal_syn6	Primer extension PCR	pGal_syn1_6_for	TTTGAAGACAAGGAGTTAGAAGGCGCGGATTAG

		pGal_syn1_6_rev	TTTGAAGACACCATTTGTTTTTTTTTCTTG ACGTTAATGT
pGal_syn7	Primer extension PCR	pGal_syn1_7_for	TTTGAAGACAAGGAGTGGTTGTGGTCGGA TTAGAA
		pGal_syn1_7_rev	TTTGAAGACACCATTTTTTTTTTCTCTTTG ATGTTAACGT

Supplementary Table 2: Sequences of synthetic galactose inducible promoters and assignment to their assembled genes and terminators.

**Promoter**		**Gene**	**Terminator**	
**Name**	**Sequence**		**Name**	**Source**
pGal_ syn1	ATTACTATCCCGGATTAGAAGCCGCCGC ACGGGCGACAGCCCTCCGAAACGCGCC GCACTGCTCCGAACAATGGTTCTCGTGC TGTTGCTGACACCGGCGGTCTTTCGTCC GTGCCTTCGACGTGAGACTTCAACTATA TAAATGCAAAAACTGTATAAAAACTTTA ACTAATACTTTCAACATTTTCGGTTTGT ATTGCTCCTCATTCACATCTTATTCAATT ATCATCAAAAAATTGTTAATATACCTCT ATAGCTTAACGTCATAAAAAAAAAACA	*GGPPS*	tASP3-1	[1]
pGal_ syn3	AGAGTGTGGTCGGATTAGAAGCCGCCG CACGGGCGACAGCCCTCCGACCCGCGC	*MiS*	tSAG1	[1]

	CGCACTGCTCCGAACAATAGGGGTTGA GAAAGCCGGCCCACCGGCGGTCTTTCGT CCGGCCAAGTTCCACTTGTCCGGTCTAT ATAAATGCAAAAACTGCATATAAACTTT AACTAATACTTTCAACAATTTCGGTTTG TATTGCTCCTCATTCTCATCTTATTAAAG TATCATCAAGAAATTGTTAATATACCTC TATACATTAACATCATAAAAAAAAAAC A			
pGal_ syn4	TTGTGTTGTTCGGATTAGAAGCCGCCGA CCGGGCGACAGCCCTCCGACTCGCGCC GCACTGCTCCGAACAATCTGGAGATGC ACTTGTGGCCCACCGGCGGTCTTTCGTC CGTGCGGCCTGTTGCGTAGCAGAGCTAT ATAAATGCAAAAACTGCATATCCACTAT AACTAATACTTTCAACAATTTGGTGTTG TATTGCTTTTCATTCTAATGTTATTCAAG TATCATCAAGAAATTGTTAATATACCTC TATAGCTTAACGTCAAAGAAAAAAAAC A	*ATR1*	tALY2	[1]
pGal_ syn5	GGCTTAGCGTCGGATTAGAAGCCGCCGT ACGGGCGACAGCCCTCCGAACCGCGCC GCACTGCTCCGAACAATCTGCGCCAAA	$C_{20}Ox$	tCYC1	[2]

	AAGGGCGTGGCACCGGCGGTCTTTCGTC<br>CGTGCCTTCCTTTGTAGATTAAGTCTAT<br>ATAAATGCAAAAACTGCATAACCACTA<br>TAACTAATACTTTCAACAATTTCGGGTT<br>GTATTACTCTTTATTCTCATCTTATAAAA<br>GTATCAACAAGAAATAGTTAATATACCT<br>CTATACCTTAACATCATAAAAAAAAAA<br>AA			
pGal_<br>syn6	TTAGAAGGCGCGGATTAGAAGCCGCCG<br>CGCGGGCGACAGCCCTCCGACGCGCGC<br>CGCACTGCTCCGAACAATTCTATTAGAC<br>GCTCAGTTATCACCGGCGGTCTTTCGTC<br>CGTGCAGGATGGAGTAAATGTAGACTA<br>TATAAATGCAAAAACTGTATAACAACTT<br>TAACTAATACTTTCAACAATTTCGTGTT<br>GTATTGCTTCTCATTCTCACGTTATTAAA<br>GTATCATCAAAAAATAGTTAATATACCT<br>CTATACATTAACGTCAAGAAAAAAAAA<br>CA	*(H)FS*	tPGK1	[1]

pGal_ syn7	TGGTTGTGGTCGGATTAGAAGCCGCCGT ACGGGCGACAGCCCTCCGATTCGCGCC GCACTGCTCCGAACAATTAGGTCGAGTT GGGTCCGGTCACCGGCGGTCTTTCGTCC GTGCTCCGACACTGGACTCTCCACTATA TAAATGCAAAAACTGTATATCAACAAT AACTAATACTTTCAACATTTTCGGTTTG TATTGCTCCTCATTCAAATCTAATTAAA GTATCATCAAGAAATTGTTAATATACCT CTATACGTTAACATCAAAGAGAAAAAA AA	*CPS*	tDIT1	[1]

Supplementary Table 3: Constructs and corresponding yeast strains.

Strain	Transformed gene combination	Source
	GGPPS:CPS:MiS (control)	[3]
	GGPPS:CPS:MiS:ATR1:CYP76AH1	[3-5]
	GGPPS:CPS:MiS:ATR1:CYP76AH22	[3,4,6]
	GGPPS:CPS:MiS:ATR1:CYP76AH23	[3,4,6]
	GGPPS:CPS:MiS:ATR1:CYP76AH4	[3,4,7]
	GGPPS:CPS:MiS:ATR1:CYP76AH24	[3,4,6]
	GGPPS:CPS:MiS:ATR1:CYP76AH22:CYP76AK6	[3,4,6]
	GGPPS:CPS:MiS:ATR1:CYP76AH23:CYP76AK6	[3,4,6]
	GGPPS:CPS:MiS:ATR1:CYP76AH24:CYP76AK6	[3,4,6]
**INV*Sc*1**	*GGPPS:CPS:MiS:ATR1:CYP76AH4:CYP76AK6*	[3,4,7]
	GGPPS:CPS:MiS:ATR1:CYP76AH1:CYP76AK6	[3-5]
	GGPPS:CPS:MiS:ATR1:CYP76AH22:CYP76AK7	[3,4,6]
	GGPPS:CPS:MiS:ATR1:CYP76AH23:CYP76AK7	[3,4,6]
	GGPPS:CPS:MiS:ATR1:CYP76AH24:CYP76AK7	[3,4,6]
	GGPPS:CPS:MiS:ATR1:CYP76AH4:CYP76AK7	[3,4,7]
	GGPPS:CPS:MiS:ATR1:CYP76AH1:CYP76AK7	[3-5]
	GGPPS:CPS:MiS:ATR1:CYP76AH22:CYP76AK8	[3,4,6]
	GGPPS:CPS:MiS:ATR1:CYP76AH23:CYP76AK8	[3,4,6]
	GGPPS:CPS:MiS:ATR1:CYP76AH24:CYP76AK8	[3,4,6]

GGPPS:CPS:MiS:ATR1:CYP76AH4:CYP76AK8	3,4,7
GGPPS:CPS:MiS:ATR1:CYP76AH1:CYP76AK8	3-5

CYP76AH22, CYP76AH23 and CYP76AH24 were formerly known as RoFS1, RoFS2 and SfFS, respectively.

Supplementary Table 4: NMR data of 11-hydroxyferruginol and hydroxyferruginol quinone. (^{13}C: δ [ppm], ^{1}H: δ [ppm] m (J [Hz]) ($CDCl_3$) obtained from a Agilent VNMRS 600, $CDCl_3$, ^{1}H @ 599.829 MHz (^{1}H; ^{1}H,^{13}C HSQC; ^{1}H,^{13}C HMBC), reference: ^{1}H: tetramethylsilane int. = 0 ppm; concentration: ca. 0.2 mmol/L. Carbon positions refer to Fig. S5A.

	11-Hydroxyferruginol			Hydroxyferruginol quinone	
**Pos.**	**^{13}C^{a}**	**^{1}H**	**HMBC (H to C)**	**^{13}C^{a}**	**^{1}H**
1	36.8	3.043 dt-like (13.3/3.5)/ 1.33[a]		35.9	2.723 dt-like (13.4/3.6)/ n.d.
2	n.d.	n.d.		n.d.	n.d.
3	41.4	1.46[a]/1.23[a]		n.d.	n.d.
4	33.7	---		n.d.	---
5	52.9	1.31[a]		51.5.	n.d.
6	n.d.	n.d.		n.d.	n.d.
7	32.4	2.79[a]/2.79[a]	5, 8, 9, 14	33.8 (?)	2.474 ddd (20.6/6.1/1.1)/

					2.406 ddd (20.6/10.8/7.1)
8	129.6	---		n.d.	---
9	133.0	---		144.9	---
10	39.2	---		38.0	---
11	142.9	---		n.d.	---
12	138.1	---		n.d.	---
13	131.4	---		n.d.	---
14	117.2	6.437 s	7, 9, 12, 15,	137.8	6.384 s
15	27.1	2.983 sp (6.9)	12, 13, 14, 16, 17	26.9	2.897 sp (7.1)
16[b]	22.4	1.250 d (6.9)	13, 15, 17	21.5	1.093 d (7.0)
17[b]	22.7	1.230 d (6.9)	13, 15, 16	21.5	1.087 d (7.0)
18	21.9	0.926 s	3, 4, 5, 19	21.7	0.884 s
19	33.7	0.952 s	3, 4, 5, 19	33.5	0.926 s
20	20.2	1.335 s	1, 5, 9, 10	20.0	1.224 s
11-OH	---	5.622 s	9, 11, 12	---	---
12-OH	---	4.577 s	11, 12, 13	---	---

[a] chemical shifts of HSQC or HMBC correlation peaks; [b] may be interchanged; n.d. not detected; s singlet; d doublet; t triplet; sp septet; ddd doublet of doublet of doublet.

Supplementary Table 5: NMR data of miltiradien-20-al. (^{13}C: δ [ppm], ^{1}H: δ [ppm] m (J [Hz]) ($CDCl_3$) obtained from a Agilent VNMRS 600, C_6D_{12}, ^{1}H @ 599.829 MHz (^{1}H; ^{1}H,^{13}C HSQC; ^{1}H,^{13}C HMBC), reference: ^{1}H: tetramethylsilane int. = 0 ppm. Carbon positions refer to Fig. S5A.

**Miltiradien-20-al**			
**Pos.**	**$^{13}C^{a}$**	**^{1}H**	**HMBC (H to C)**
1	30.3	2.49[a] {β} / 0.78[a] {α}	
2	19.7	1.555 tt-like (13.7/3.6) {β} / 1.42[a] { α }	
3	42.3	1.37[a] {β} / 1.17 { α }	
4	34.0	---	
5	52.0	1.1.506 dd (13.1/2.8)	4, 6, 10, 18, 19, 20
6	18.0	2.06[a] {β} /<br>1.86[a] { α }	5, 7, 10<br>5, 7, 8, 10
7	32.0	2.17[a] { α } / 2.09[a] {β}	
8	132.8	---	
9	123.6	---	
10	53.6	---	
11	25.5	2.43[a] / 2.24[a]	
12	116.7	5.343 m	11, 14, 15
13	138.8	---	
14	34.8	2.60[a] /<br>2.47[a]	8, 9, 12, 13<br>8, 9, 12, 13

15	34.8	2.13[a]	12, 13, 14, 16, 17
16	21.1	0.998[b] d (6.9)	13, 15, 17
17	21.1	0.994[b] d (6.9)	13, 15, 16
18	20.6	0.789 s	3, 4, 5, 19
19	31.6	0.920 s	3, 4, 5, 18
20	194.8	9.599 br d (1.8) {$^{1}J_{CH}$ = 181 Hz}	1, 10

[a] chemical shifts of HSQC or HMBC correlation peaks; [b] may be interchanged;

s singlet; d doublet; t triplet; { } additional information.

Supplementary Table 6: NMR data of carnosaldehyde. (^{13}C: δ [ppm], ^{1}H: δ [ppm] m (J [Hz]) ($CDCl_3$) obtained from a Agilent VNMRS 600, $CDCl_3$, ^{1}H @ 599.829 MHz (^{1}H; ^{1}H,^{13}C HSQC; ^{1}H,^{13}C HMBC), reference: ^{1}H: tetramethylsilane int. = 0 ppm. Carbon positions refer to Fig. S5A.

**Carnosaldehyde**			
**Pos.**	**^{13}C[a]**	**^{1}H**	**HMBC (H to C)**
1	30.5	3.249 dt-like (13.5/4.2) {β} / 1.15[a] {α}	
2	19.7	1.58[a] {α} / 1.54[a] {β}	
3	41.3	1.49[a] {β} / 1.330 td-like (13.2/4.8) { α }	
4	34.2	---	
5	52.9	1.62[a]	20

6	18.8	2.029 br d (12.7) { α } /<br>1.863 m {β}	
7	31.5	2.866 dd-like (8.5/3.5) {2H}	5, 6, 8, 9, 14
8	129.9	---	
9	116.2	---	
10	n.d.	---	
11	143.4	---	
12	142.3	---	
13	134.5	---	
14	119.4	6.599 s	7, 9, 12, 15,
15	27.0	3.234 sp (6.9)	12, 13, 14, 16, 17
16	22.2	1.209 d (7.0)	13, 15, 17
17	22.2	1.209 d (7.0)	13, 15, 16
18	21.5	0.903 s	3, 4, 5, 19
19	31.9	1.039 s	3, 4, 5, 18
20	203.5	9.901 s {$^{1}J_{CH}$ = 175 Hz}	1, 5
11-OH	---	7.129 s	11, 12
12-OH	---	5.786 s	11, 12, 13

[a] chemical shifts of HSQC or HMBC correlation peaks; n.d. not detected; s singlet,; d doublet; t triplet; sp septet; { } additional information.

Supplementary Table 7: Protein sequences used for phylogenetic analysis with their corresponding accession numbers.

Protein	Species	Accession number
AaCYP71AV1	*Artemisia annua*	BAM68808
AtCYP76C1	*Arabidopsis thaliana*	AT2G45560
AtCYP76C2	*Arabidopsis thaliana*	AT2G45570
AtCYP76C4	*Arabidopsis thaliana*	AT2G45550
BsGAO	*Barnadesia spinosa*	GU256647
CaCYP76B4	*Camptotheca acuminata*	AES93118
CfCYP71D381	*Coleus forskohlii*	Patent WO/2015/113569
CfCYP76AH11	*Coleus forskohlii*	Patent WO/2015/113569
CfCYP76AH15	*Coleus forskohlii*	Patent WO/2015/113569
CfCYP76AH17	*Coleus forskohlii*	Patent WO/2015/113569
CfCYP76AH8	*Coleus forskohlii*	Patent WO/2015/113569
CfCYP76AH9	*Coleus forskohlii*	Patent WO/2015/113569
CiGAO	*Cichorium intybus*	GU256644
CrCYP76B6	*Catharanthus roseus*	AJ251269
CrCYP76T24[a]	*Catharanthus roseus*	KF302075
HaGAO	*Helianthus annuus*	GU256646
HtCYP76B1	*Helianthus tuberosus*	Y09920
LsCYP71BL2	*Lactuca sativa*	F8S1I0
LsGAO	*Lactuca sativa*	GU198171
MgCYP71D18	*Mentha x gracilis*	Q6WKZ1

MgCYP76AH2[a]	*Mimulus guttatus*	1.1978 (http://drnelson.uthsc.edu/ CytochromeP450.html)
MgCYP76AH9[a]	*Mimulus guttatus*	14.23151 (http://drnelson.uthsc.edu/ CytochromeP450.html)
MpCYP71D13	*Mentha x piperita*	Q9XHE7
NtCYP71D20	*Nicotiana tabacum*	Q94FM7
OsCYP76M7	*Oryza sativa*	AK105913
PaCYP71A1	*Persea americana*	AAA32913
PhCYP76A3	*Petunia x hybrida*	BAC53891
PhCYP76A4	*Petunia x hybrida*	AB016061
RoCYP76AH22	*Rosmarinus officinalis*	KP091843
RoCYP76AH23	*Rosmarinus officinalis*	KP091844
RoCYP76AH4	*Rosmarinus officinalis*	DOI: 10.1039/c000000x/
RoCYP76AK7	*Rosmarinus officinalis*	KX431219
RoCYP76AK8	*Rosmarinus officinalis*	KX431220
SaCYP76F37v1	*Santalum album*	KC533717
SaCYP76F38v1	*Santalum album*	KC533715
SaCYP76F39v1	*Santalum album*	KC533716
ScGAO	*Saussurea costus*	GU256645
SfCYP76AH24	*Salvia fruticosa*	KP091842
SfCYP76AK6	*Salvia fruticosa*	KX431218
SmCYP76AH1	*Salvia miltiorrhiza*	AGN04215
SmCYP76B4	*Swertia mussotii*	D1MI46

SmCYP76S7[a]	*Salvia miltiorrhiza*	KP337691
VcCYP76AB1	*Vanda coerulea*	ACC59773
ZzCYP71BA1	*Zingiber zerumbet*	BAJ39893

[a] not characterized yet

Supplementary References

1 Yamanishi, M. *et al.* A Genome-Wide Activity Assessment of Terminator Regions in *Saccharomyces cerevisiae* Provides a "Terminatome" Toolbox. *ACS Synthetic Biology* **2**, 337-347, (2013).

2 Zaret, K. S. & Sherman, F. DNA sequence required for efficient transcription termination in yeast. *Cell* **28**, 563-573 (1982).

3 Brückner, K. *et al.* Characterization of two genes for the biosynthesis of abietane-type diterpenes in rosemary (*Rosmarinus officinalis*) glandular trichomes. *Phytochemistry* **101**, 52-64 (2014).

4 Mizutani, M. & Ohta, D. Two Isoforms of NADPH:Cytochrome P450 Reductase in *Arabidopsis thaliana*: Gene Structure, Heterologous Expression in Insect Cells, and Differential Regulation. *Plant Physiology* **116**, 357-367, (1998).

5 Guo, J. *et al.* CYP76AH1 catalyzes turnover of miltiradiene in tanshinones biosynthesis and enables heterologous production of ferruginol in yeasts. *Proc Natl Acad Sci U S A* **110**, 12108-12113, (2013).

6 Božić, D. *et al.* Towards Elucidating Carnosic Acid Biosynthesis in Lamiaceae: Functional Characterization of the Three First Steps of the Pathway in *Salvia fruticosa* and *Rosmarinus officinalis*. *PLoS One* **10**, e0124106, (2015).

7 Zi, J. & Peters, R. J. Characterization of CYP76AH4 clarifies phenolic diterpenoid biosynthesis in the Lamiaceae. *Org Biomol Chem* **11**, 7650-7652 (2013).
